# Changes in the Expression of Renal Brush Border Membrane *N*-Glycome in Model Rats with Chronic Kidney Diseases

**DOI:** 10.3390/biom11111677

**Published:** 2021-11-11

**Authors:** Aiying Yu, Jingfu Zhao, Shiv Pratap S. Yadav, Bruce A. Molitoris, Mark C. Wagner, Yehia Mechref

**Affiliations:** 1 Department of Chemistry and Biochemistry, Texas Tech University, Texas City, TX 79409, USA; aiying.yu@ttu.edu (A.Y.); Jingfu.zhao@ttu.edu (J.Z.); 2 Nephrology Division, Department of Medicine, Indiana University, Indianapolis, IN 46202, USA; ssyadav@iu.edu (S.P.S.Y.); bmolitor@iu.edu (B.A.M.); wagnerm@iu.edu (M.C.W.)

**Keywords:** glycomics, brush-border membrane, chronic kidney disease, proteinuria and hypertension, obese and diabetic, LC-MS/MS

## Abstract

Chronic kidney disease (CKD) is defined by a reduced renal function i.e., glomerular filtration rate (GFR), and the presence of kidney damage is determined by measurement of proteinuria or albuminuria. Albuminuria increases with age and can result from glomerular and/or proximal tubule (PT) alterations. Brush-border membranes (BBMs) on PT cells play an important role in maintaining the stability of PT functions. The PT BBM, a highly dynamic, organized, specialized membrane, contains a variety of glycoproteins required for the functions of PT. Since protein glycosylation regulates many protein functions, the alteration of glycosylation due to the glycan changes has attracted more interests for a variety of disease studies recently. In this work, liquid chromatography-tandem mass spectrometry was utilized to analyze the abundances of permethylated glycans from rats under control to mild CKD, severe CKD, and diabetic conditions. The most significant differences were observed in sialylation level with the highest present in the severe CKD and diabetic groups. Moreover, high mannose *N*-glycans was enriched in the CKD BBMs. Characterization of all the BBM *N*-glycan changes supports that these changes are likely to impact the functional properties of the dynamic PT BBM. Further, these changes may lead to the potential discovery of glycan biomarkers for improved CKD diagnosis and new avenues for therapeutic treatments.

## 1. Introduction

Glycosylation is one of the most important post-translational modifications (PTMs) of proteins, and it plays an essential role in numerous biological processes such as immune response, cell-extracellular communication, and cellular metabolism [1,2,3,4]. Nearly all membrane proteins and intracellular proteins are modified by oligosaccharides, which makes glycosylation a valuable source of biofunctional information and disease research [5]. The microheterogeneity of glycan structures is complex due to many features, such as various monosaccharide residue compositions and the diversity of linkages and branches. This is one of the factors that causes the alteration of the glycosylation which has been associated with many diseases including cancers [1,6,7,8,9,10,11], Alzheimer’s disease [12,13], and diabetes [14,15,16].

The PTs contain two unique plasma membrane domains: an apical surface with microvilli and a basolateral domain each with distinct proteins and lipid compositions. This highly polar spatial organization is necessary for physiological functions and dependent upon the correct sorting and delivery of proteins and lipids to their respective surfaces [17,18]. Multiple vectorial transport processes depend upon this organization which leads to the formation of urine from the glomeruli filtrate [19]. For over 40 years many laboratories have used a divalent cation procedure to purify the apical microvilli brush border membranes (BBMs). The use of purified BBMs from renal proximal tubules has contributed significantly to the understanding of transepithelial solute transport mechanisms. Although many of the BBM proteins are glycoproteins, very little information exists on kidney glycomics changes with diseases. In this study, we focused on documenting glycomics changes in the purified BBM that occur with CKD and diabetes.

Chronic kidney disease (CKD) is defined as an untreatable and progressive disease process that reduces kidney function and causes kidney damage [5]. The global prevalence of CKD is estimated to be 8–16% and it was reported to affect up to 10% of the US population [20,21]. CKD is defined by a reduced renal function i.e., reduced glomerular filtration rate (GFR) and the presence of kidney damage is also determined by measurement of proteinuria or albuminuria. Proteinuria, increases with age and can result from glomerular and/or PT alterations [22]. The quest for using prognostic biomarkers to predict CKD and its progression has received interest in recent years. Innovative technologies such as proteomics, peptidomics, and functional genomics have discovered promising biomarkers for CKD diagnosis [23,24,25,26]. Such biomarkers include asymmetric dimethylarginine, symmetric dimethylarginine, uromodulin, kidney injury molecule-1, and neutrophil gelatinase-associated lipocalin. Although many studies have been reported at the protein level to discover potential biomarkers for CKD, glycomics profiling on glycoproteins has not been well-documented. Moreover, evidence has shown that the steady rise in CKD prevalence may be associated with increasing proteinuria, hypertension, and obesity [27,28,29], although less attention has been paid to the links between proteinuria, hypertension, and obesity with CKD. Glycomics profiling of BBM glycoproteins purified from control rat kidneys and those with distinct diseases will contribute to a more complete understanding of the dysfunction and may identify glycan biomarkers for CKD diagnosis.

Mass spectrometry (MS) is one of the most common analytical techniques that can provide reliable identification capability for glycomics studies [30,31]. Collision-induced dissociation (CID), one of the fragmentation techniques of MS, has been widely applied for generations of oligosaccharide tandem mass spectra for detailed structural information [32,33]. MS coupled with liquid chromatography (LC) is a powerful analytical method for comprehensive qualitative, and quantitative analysis of glycans because of its high throughput, high sensitivity, and its capability for complex sample analysis. However, the quantitation of native glycans can be problematic due to the low ionization efficiency. In addition, the possible sialic acid loss and fucose migration during ionization often hinders structural assignments of glycans [34,35]. To stabilize glycan structures and improve ionization efficiency during fragmentation, permethylation is a common method that is used to derivatize glycans [36]. Permethylated glycans contribute to higher ionization efficiency in positive mode by increasing proton affinity, which helps to achieve higher sensitivity. Permethylation can also improve the fragmentation pattern of glycans in MS/MS analysis, resulting in more reliable glycomics profiling.

In this work, five distinct rat models were used to isolate BBMs. *N*-glycans released from these BBMs were quantitatively profiled by sensitive C18 LC-MS/MS. C18 reverse-phase liquid chromatography (RPLC) has been widely used for glycomics profiling in academic and industrial laboratories due to its extraordinary reproducibility and reliability in quantitation. By comparing BBM glycomics profiles from healthy, proteinuric, hypertensive and diabetic rats, it will better define the alterations contributing to these physiological states and may reveal specific biomarkers for improved therapeutic kidney treatments. 

## 2. Materials and Methods

### 2.1. Materials and Reagents

Peptide: *N*-glycosidase F (PNGase F) was obtained from New England Biolabs (Ipswich, MA, USA). Ammonium bicarbonate (ABC), sodium deoxycholate (SDC), ammonium-borane complex, iodomethane, dimethyl sulfoxide (DMSO), and sodium hydroxide (NaOH) beads were purchased from Sigma-Aldrich (St. Louis, MO, USA). Anhydrous ethanol was obtained from Pharmco-Aaper. High performance liquid chromatography (HPLC) grade methanol, acetonitrile (ACN), formic acid (FA), and HPLC water were obtained from Fisher Scientific (Fair Lawn, NJ, USA). 

### 2.2. Animals

Munich Wistar Frömter (MWF) rats were derived from a colony generously provided by Dr. Roland Blantz (UCSF, San Diego, CA, USA) and maintained in the Indiana University LARC facility. Zucker ZSF1 obese male rats were obtained from Charles River. All rats received water and food (MWF received standard rat chow and ZSF1 received LabDiet 5008) ad libitum throughout the study. The Purina #5008 diet is recommended for the male obese ZDF rats to induce programmed and consistent development of Type 2 diabetes (Charles Rivers Labs). Since we wanted to evaluate a diabetic model as a model with documented increased and altered glycosylation, we maintained their normal diet which is the #5008. All experiments followed NIH Guide for the Care and Use of Laboratory Animals guidelines and were approved by the Animal Care and Use Committee at the Indiana University School of Medicine.

### 2.3. Isolation of Renal Proximal Tubular Brush-Border Membranes

The procedure of isolation and purification of BBMs was adapted from the mouse kidney procedure by Biber et al. [19]. Briefly, rats were perfused with saline, kidneys removed, and outer cortex dissected in cold PBS followed by rapid freezing in liquid nitrogen. Homogenization consisted of placing 1 g tissue into 7.5 mL cold buffer A (300 mM mannitol, 5 mM egtazic acid (EGTA), 12 mM Tris-base, pH 7.1, 1 mM phenylmethylsulfonyl fluoride, and protease cocktail). The cortex was homogenized using the Polytron tip PTA10S (2–45 s bursts). Next, 10 mL water and 210 µL of 1 M magnesium chloride (21 mM final concentration) were added to the tube, mixed, and placed on ice for 15 min. Tubes were then centrifuged at 3000 *g* for 15 min at 4 °C which resulted in a pellet. Supernatant was then centrifuged at 38,000 *g* for 30 min at 4 °C. The pellet was resuspended with buffer B (150 mM mannitol, 2.5 mM EGTA, 6 mM Tris-base, pH 7.1) and centrifuged at 21,000 *g* for 30 min. Two cycles of Mg precipitation were conducted, and the final BBM pellet were resuspended in buffer B, added to microfuge tubes, pelleted, froze in liquid nitrogen, and stored at −80 °C till analysis.

### 2.4. Protein Extraction

The steps for extracting proteins are as follows: BBM pellets were thawed at room temperature. 100 µL of the ABC buffer and 100 µL 5% SDC buffer were added. Next, the BBM was homogenized at 4 °C using a beads beater (BeadBug microtube homogenizer, Benchmark Scientific, Edison, NJ, USA). The procedure was 30 s shaking followed by 30 s stop with a total of 5 cycles. After that, samples were further broken up by sonicating in ice for 1 h. Next, samples were centrifuged at 1000 *g* for 1 min and then remained at 14,800 *g* for 10 min. Finally, supernatants were collected and transferred to new Eppendorf tubes.

### 2.5. N-Glycan Release and Purification

Proteins were denatured at 90 °C for 15 min and 2 µL proteins were used for protein assay, which was performed by a BCA protein assay kit (Thermo Scientific/Pierce, Rockford, IL, USA). The protein concentration was measured by a Multikan plate reader (Thermo Scientific, Rockford, IL, USA). After the protein measurement, 50 µg of proteins were digested with PNGase F in a 37 °C water bath for 18 h. The ratio between the protein amount (µg) and the units of PNGase F was 1:3. After digestion, 0.1% FA was added to remove SDC. Then, samples were centrifuged for 10 min at 14,800 *g* and the supernatant was collected. Released *N*-glycans in the supernatant were dried using a SpeedVac benchtop vacuum concentrator (Labconco, Kansas City, MO, USA). 90% ethanol was added to extract glycans and centrifugation was performed to remove pellets. The supernatant was collected and dried again. Glycans were resuspended with 50 µL of water and subjected to dialyzation to remove salts and the remaining SDC using a 500–1000 molecular weight cutoff dialysis membrane (Spectrum Laboratories, Rancho Dominguez, CA, USA). After dialysis, purified *N*-glycans were dried for the following reduction and solid-phase permethylation.

### 2.6. Reduction of Glycans

The *N*-glycan reduction procedure aims to eliminate the α and β anomers from the reducing end of the released glycans. Glycans were reduced as described previously [37]. Briefly, 10 μL of the borane-ammonia complex solution (10 mg/mL) was mixed with the *N*-glycans. The mixture was incubated in a 60 °C water bath for 1 h. After the reaction finished, 500 μL of methanol was added to the reduced *N*-glycans and dried; the methanol reacted with extra ammonia-borane to form volatile methyl borate salts [36]. This glycan purification step was repeated 3 to 4 times to remove any remaining ammonia-borane complex and borate salt until the samples were dried.

### 2.7. Solid-Phase Permethylation of N-Glycans

The reduced *N*-glycans were permethylated using the solid-phase permethylation method as previously described [36,37]. Dried samples were dissolved in 30 μL DMSO and 1.2 μL HPLC water. Sodium hydroxide beads that were suspended in DMSO were loaded onto the spin column, and centrifuged at 1800 *g* for 2 min. Then 200 μL of DMSO was added to wash the column followed by a 2nd centrifugation at the same speed. Next, 20 μL iodomethane was added to the dissolved glycans and vortexed. This mixture was loaded onto the spin column and incubated for 25 min. Another 20 μL iodomethane was then added to the spin column and incubated for another 15 min. After incubation, the spin columns were centrifuged at a speed of 1800 *g* for 2 min. To elute the permethylated glycans, 30 μL ACN was added to the columns followed by centrifugation at 1800 *g* for 1 min. Eluents were dried overnight under vacuum. Dried permethylated glycans were resuspended with 20% ACN and 0.1% FA for LC-MS/MS analysis.

### 2.8. LC-MS/MS Analysis

Permethylated glycans were analyzed with the Dionex 3000 UltiMate NanoLC system (Dionex, Sunnyvale, CA, USA) coupled with a LTQ Orbitrap Velos mass spectrometer (Thermo Scientific, San Jose, CA, USA). Each injection contained *N*-glycans released from 50 µg proteins. In the LC system, a reverse-phase C18 trap (Acclaim PepMap 100, 75 µm × 2 mm, 3 µm, 100 Å, Thermo Scientific) was used to remove salts and impurities. After purification, *N*-glycans were separated using a C18 column (Acclaim PepMap 100, 75 μm × 150 mm, 2 μm, 100 Å, Thermo Scientific). The flow rate was 0.35 μL/min. Mobile phase solvent A contained 98% water, 0.1% FA, and 2% ACN while mobile phase B consisted of 100% ACN and 0.1% FA. 20% mobile phase B was used in the first 10 min, then increased to 42% at 11 min. Mobile phase B continued to linearly increase to 55% from 11 to 48 min and increased to 90% at 49 min. The gradient remained at 90% B for the next 5 min, then dropped to 20% B from 55 to 60 min.

The LTQ Orbitrap Velos mass spectrometer was set to the positive mode with three scan events. The first was a full MS scan with a *m*/*z* range of 700 to 2000 which acquired at 100,000 resolution. The second and third events were data-dependent MS/MS scans, namely CID and HCD (higher-energy collision dissociation) MS/MS. The top four most intense ions were selected through data-dependent acquisition and then subjected to CID and HCD MS/MS. Dynamic exclusion parameters were set as follows: repeat count 2; repeat duration 30 s. This set-up enabled us to exclude ions repeated within 30 s. The normalized energy used for CID was 35%, and 45% for HCD. The isolation width was 3.0 *m*/*z* for both, and the activation time was 15 ms for CID and 0.1 ms for HCD. 

Multiple-reaction monitoring (MRM) was performed in TSQ Vantage triple quadrupole mass spectrometer (Thermo Scientific). MRM LC-MS/MS has been used as a rapid and reliable identification and quantification method for permethylated *N*-glycans [38]. In this study, it was used as an auxiliary method to confirm the glycan structures. Parameters for MRM method were set as follows: the peak width was set at 0.7 FWHM, and scan time was 0.7 s at mass range 500–1500 *m*/*z*. Normalized collision energy was set at 30% to 45%. 

### 2.9. Data Analysis

Raw files of each sample group were processed by the open-source MultiGlycan software to detect possible glycan structures followed by a manual check [39]. This software can provide abundance information of detected glycans based on the ion intensities of the glycan with all charge states and adducts through MS scans. The confirmation of *N*-glycan structures was based on checking the full MS and MS^2^ manually using Xcalibur (Thermo Scientific, Version 4.2) software. The quantitative analysis of the *N*-glycans was based on peak area. To profile *N*-glycans in the BBM samples, a relative quantitation of *N*-glycans was utilized to investigate glycan expressions [40]. The relative abundance of each identified *N*-glycan was calculated by dividing the individual *N*-glycan peak area by the total *N*-glycan peak area. After normalization, the relative quantitation results of *N*-glycans from each sample group were used for the unsupervised principal component analysis (PCA) by MarkerView Software (Sciex, Version 1.3). To study the variations in glycan expression from different sample groups, a two-tailed student *t*-test was performed to identify statistically significant *N*-glycan structures between the control and disease group. Moreover, the Bonferroni Correction was performed for multiple analyses to avoid Type I error.

## 3. Results and Discussion

### 3.1. Rat Kidney Cortex Brush-Border Membrane Specification

BBM pellets were isolated from the kidney cortices of five different groups of rats. Group one to four were Munich Wistar Frömter rats, which have been studied extensively and are distinguished by the development of hypertension and albuminuria by week 8 in the males which escalated progressively to ≥400 mg/24 h urinary albumin excretion by week 32, with approximately 50% sclerotic glomeruli by week 40 [41,42,43]. Note, only mild proteinuria is observed in the older females from group one. Group five was the Zucker obese male ZSF1 rats from Charles Rivers which are characterized by hypertension, dyslipidemia, and hyperglycemia, and serve as a diabetic model [44]. Table 1 contains detailed information for these five sample groups. Mild proteinuria is defined as 60–100 mg/24 h urinary albumin excretion while proteinuria was >100 mg/24 h. Normal rat systolic blood pressure is 110–120 mmHg, the rats from group three and five showed mild hypertension with 140–160 mmHg. The body weight of rats over than 300 g at 20–24 weeks is defined as obesity. The average weight of ZSF1 rats was 572 g. Hyperlipidemia is defined as elevated triglycerides and cholesterol as well as glucose >8 mmol/L. ZSF1 rats had an average triglyceride of 20 mmol/L, cholesterol of 10 mmol/L and average glucose of 22.2 mmol/L. Urine protein was measured after collecting a 24 h urine samples from each rat that had been placed in a metabolic cage. Total proteins were measured using the Lowry method. Blood pressure was measured using an AD Instruments (Colorado Spring, CO, USA) PowerLabs blood pressure transducer. Plasma triglycerides, cholesterol and glucose were measured using the Pointe 180QT semi-automatic clinical analyzer (MedTest, Canton, MI, USA). These five groups of rat models enabled us to define and compare BBM *N*-glycan profiles to better understand how glycosylation contributes to dysfunction. Several studies have shown significant *N*-glycan expression changes in sera from diabetic patients, ovarian tissues from diabetic mice, and kidney tissue from diabetic rats [15,16,45,46], while another study showed the importance of *N*-glycan moieties for the transcytosis rat FcRn receptor [47].

The use of Mg^2+^ ions to aggregate and remove non-brush border membranes was first introduced by Schmitz [48] and Booth [49] in the 1970s. Their aggregation and separation from BBM are a property of the BBM having a higher density of negative surface charges thus preventing divalent cations from forming aggregates. By performing 2 cycles of Mg^2+^ ppt, the BBM was enriched over other membranes by 10–15 folds. For assess the purity, we were starting with dissected kidney in which we perform a macroscopic dissection of the kidney cortex which ensures the starting material is mostly composed of proximal tubule segments (kidney cortex is app 90% proximal tubules). To better control for variation between BBM preparations, all kidney cortex was rapidly frozen and BBM preps performed in 3 sets each with a mix of groups. Each preparation resulted in the expected yield of protein, 1–5 mg depending upon kidney cortex size i.e., Zucker rats are very large and of course the older rats have larger kidney. The purification of BBMs was based on the study we have previously published [50], which demonstrated the successful separation of BBMs and basolateral membranes from the same homogenate. Note, we do not claim that this method yields pure BBMs but an enrichment over basolateral membranes. 

### 3.2. Establishment of BBM N-Glycan Profiling Methods

The use of purified renal brush-border membranes provides an easy and robust way to complement whole-animal studies [19]. This approach helps to investigate and analyze the functional level transport pathway and protein compositions from the proximal tubular apical membranes from experimental animal models. The PNGase F digestion method facilitated a relatively complete release of *N*-glycans from extracted proteins [51]. Permethylation, which was used to derivatize released *N*-glycans, had advantages such as higher ionization efficiency for positive ESI, increased hydrophobicity for better compatibility for RPLC separation, and increased structure stability for sialic acids [36,52]. In the LC-MS/MS system, using a C18 trap for online purification enabled a better ionization of permethylated *N*-glycans. The LTQ Orbitrap Velos mass spectrometer provided high resolution and high sensitivity for *N*-glycan detection, making the identification of *N*-glycan structures, especially for low abundance glycans, more efficient. 

A BCA protein assay was used to normalize the protein amount of each sample. After normalization, the final concentration of the initial protein from the BBM samples was identical. 50 µg of initial proteins from each sample were digested with PNGase F simultaneously under identical conditions. Then, glycans released from 50 µg of proteins were subjected to LC-MS/MS for detection of all possible glycan structures. Due to the efficient separation from LC and high sensitivity from MS, a total of 121 *N*-glycan structures were identified and quantified from 5 groups of BBM samples. This number was much higher than those found by the matrix-assisted laser desorption/ionization time of flight tandem MS methods [17,53]. The permethylation method provides enhanced ionization efficiency making the detection of low abundant glycans possible. 

Distributions of the different types of *N*-glycans among five groups were shown as pie charts in Supporting Information Figure S1. In this study, we used four-digit codes to represent *N*-glycan compositions. X-X-X-X stands for HexNAc-Hexose-DeoxyHex-NeuAc. HexNAc includes *N*-acetylglucosamine and *N*-acetylgalactosamine. Hexose includes galactose and mannose. Deoxyhexose is fucose and NeuAc is *N*-acetylneuraminic acid. Full MS and MS^2^ were used to identify *N*-glycan compositions. One example of *N*-glycan composition identification is shown in Supporting Information Figure S2. First, the composition was confirmed by the full MS, as shown in Figure S2a (inset). Further identification was achieved by confirming the diagnostic ion fragments from the *N*-glycan compositions in the MS^2^ spectrum. For example, *N*-glycan 4-5-1-1 (HexNAc_4_Hex_5_DeoxyHex_1_NeuAc_1_) has one fucose, which may be core or branch fucosylation. As opposed to branch fucosylation, core fucosylation that is connected to the reducing end of a glycan has a specific diagnostic ion at *m*/*z* 468. As shown in the MS^2^ spectrum (Figure S2b), a *m*/*z* 468 fragment ion with a green frame was detected, indicating that this structure had a core fucose. All identified *N*-glycan structures are displayed in Supporting Information Table S1. This table includes the theoretical *m*/*z*, observed *m*/*z*, average relative abundance, and standard deviation of all glycan structures from each sample group. Significant *N*-glycans (*p* < 0.05) from disease-related groups and controls were listed in Supporting Information Table S2. Moreover, a student *t*-test was performed on the relative abundance of each *N*-glycan structure. The mass accuracy of each structure was within 5 ppm.

### 3.3. Unsupervised PCA

After a thorough evaluation of *N*-glycomics methods, we applied these methods to 20 BBM samples which were derived from the five groups (*n* = 4) as shown in Table 1. Proteomic studies have been done on urine or plasma samples to reveal changes of proteomics classifiers on patients with hypertension, proteinuria, and diabetes [54,55]. However, glycomics studies of biological samples related to proteinuria, hypertension, obesity, and diabetes are seldom reported. Our work performed glycomics profiling on BBMs from control and disease animals with these chronic medical conditions to investigate the variant *N*-glycan expressions. Considering that glycoproteins from rats may vary due to gender, we compared the *N*-glycan expression within same-gender groups to avoid gender bias. Previous *N*-glycosylation studies have shown altered fucosylation *N*-glycans in serum samples from Type I diabetic patients and increased sialylated *N*-glycans in the vitreous fluid of diabetic patients [56,57]. The different expressions of fucosylated and sialylated glycans were observed in disease-related groups compared to the controls in this study. In addition, glycosylation differences of BBM proteins due simply to gender may also be present. 

**Figure 1:**
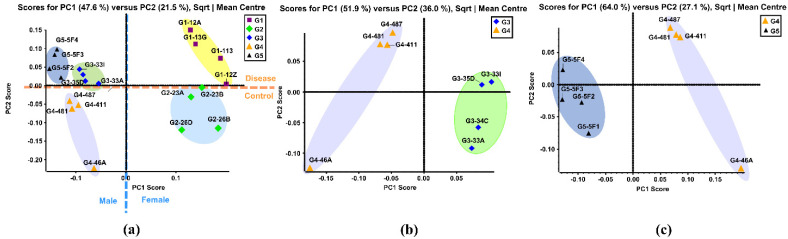
(**a**) Unsupervised PCA of the BBMs *N*-glycans from five sample groups. Symbols with same color and shape were the replicates from same sample group. (**b**) Unsupervised PCA of the BBM *N*-glycans from old male with proteinuria and hypertension (G3) and young male control (G4). (**c**) Unsupervised PCA of the BBM *N*-glycans from male control (G4) and obese and diabetic male (G5). G3 were MWF male rats (32–42 weeks), G4 were MWF male rats (7 weeks), and G5 were ZSF1 male rats (16 weeks).

PCA is a multivariate technique that can extract crucial information from complex datasets and interpret the information with a set of new orthogonal variables called principal components. This reduces the dimensionality of a complex dataset and increases interpretability while minimizing information loss. PCA plots display points in maps to show the similarity of the observations and the variables [58]. Figure 1a shows the unsupervised PCA plot that was generated using the quantitative results of 121 identified *N*-glycans of the five sample groups. Four replicates from the same sample group were clustered with the same color. Close-clustered data points indicate that replicates were reproducible and reliable. When the *N*-glycan data of five groups was plotted, five clusters were distinguishable through the primary component (PC1) and second component (PC2) without any overlap. This result indicated that there were different expressions of *N*-glycans among these five groups. 

The Group 1 (G1) cluster was close to the Group 2 (G2) cluster, representing the similarity between these two groups. This result was expected since these two were both female groups. The same result was observed in Group 3 (G3), Group 4 (G4) and Group 5 (G5), which were all male groups. The female groups and male groups were separated by the blue dotted line in Figure 1a. Moreover, clusters of disease related G1, G3, and G5 were located on the positive PC2 score, while clusters of control G2 and G4 lay on the negative PC2 score. This demonstrated that distinct differences in *N*-glycan expressions existed between disease-related groups and the controls. Figure 1b displays the PCA plot of the quantitative glycomics data from the proteinuria and hypertension male group (G3) versus the male control group (G4), while Figure 1c is the plot for the obese and diabetic male group (G5) versus G4. The same observation was obtained between G1 and G2, as shown in Supporting Information Figure S3a. Distinct differences between the disease and control groups were observed from both PCA plots, which indicated that different *N*-glycan expressions were disease-related. Along with the comparisons mentioned above, the PCA plot between the two controls (G2 and G4) also showed distinct differences in glycan abundances (Supporting Information Figure S3b). This result suggests that glycans with differential expression may be related to gender. 

### 3.4. Comparison between Proteinuria and Hypertension Groups and Control Group

Most BBM components, including the multiligand receptors megalin and cubilin, are glycosylated through multiple regulated pathways [5]. The diversity of the glycans and their location mediates many biological processes such as cell signaling, adhesion, and communication [1,5,59,60]. In addition, protein folding, stability, and localization are dependent on protein glycosylation [61,62]. Consequently, glycan alterations are found in many diseases, including hereditary disorders, immune deficiencies, cardiovascular disease, and cancer [63,64,65,66,67,68,69,70,71,72]. In addition, multiple studies have identified mutations in the BBM glycoproteins megalin and cubilin that are associated with kidney disease often manifested by proteinuria [73,74,75,76,77,78,79,80,81,82]. Further, ligand binding of both these multiligand receptors is impacted by their glycan state which would have a direct impact on levels of proteinuria [83,84,85,86]. Unfortunately, knowledge of BBM glycosylation, including cubilin and megalin, in disease states is limited as is an understanding of the changes to the glycan regulatory pathways with disease. Since the PT BBM plays a central role in handling the glomerular filtrate and overall kidney homeostasis studying how this glycome is changing and which pathways are altered are vital for understanding the mechanism(s) of glycan dysfunction. This will lead directly to the discovery of new targets and potential therapeutics that maintain the physiological glycosylation state and reduce proteinuria in kidney disease. This study and our analysis of BBM *O*-glycan changes [87] with disease states represent the first step in further defining the significance of BBM glycans.

To study the variation in *N*-glycan expression between the sample groups with proteinuria and hypertension and the control groups, a student *t*-test was performed to identify the statistically significant *N*-glycan structures that had a *p*-value less than 0.05. Many independent statistical tests were performed to eliminate false positives. However, when many tests are run simultaneously, the probability of a significant result increases with each test run. To maintain the statistical power, we employed the Bonferroni correction which enabled us to limit the possibility of obtaining a statistically significant result while testing multiple hypotheses. By making the alpha level more stringent, the Bonferroni correction reduced the probability of type I error at a 0.1 false discovery rate [88]. With sensitive LC-MS/MS analysis, a total of 121 *N*-glycans were identified and quantified. Relative abundance was used to investigate the distribution of individual glycans among different groups. The calculation of the relative abundance was achieved by dividing the individual glycan abundance by the total glycan abundance. After a *t*-test with Bonferroni correction, the *N*-glycan *p*-values were less than 0.05.

**Figure 2:**
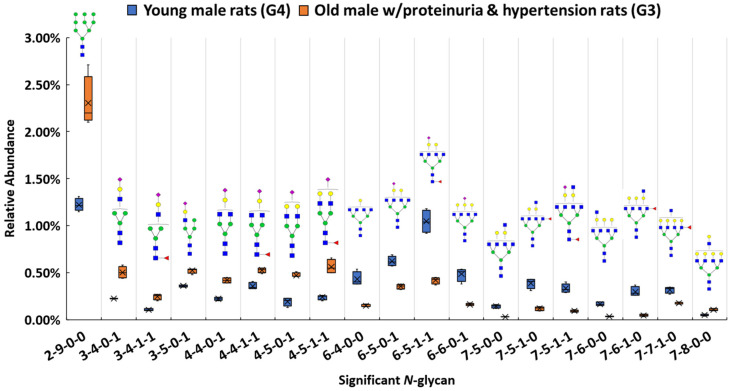
Box plot for relative abundance of 19 significant *N*-glycans (*p* < 0.0004) between old male with proteinuria and hypertension (G3) and young male control (G4). The putative structure was assigned to each glycan composition. The X axis denotes the four-digit codes for *N*-glycan compositions. The Y axis is the relative abundance. The four-digit codes represent *N*-glycan compositions. X-X-X-X stands for HexNAc-Hexose-DeoxyHex-NeuAc. HexNAc includes *N*-acetylglucosamine and *N*-acetylgalactosamine. Hexose includes galactose and mannose. Deoxyhexose is fucose and NeuAc is *N*-acetylneuraminic acid. Symbols: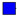, *N*-acetylglucosamine (GlcNAc);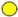, Galactose (Gal);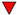, Fucose (Fuc);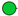, Mannose (Man);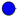, Glucose (Glc);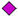, *N*-acetylneuraminic acid (NeuAc/Sialic Acid).

Among these structures, 19 *N*-glycans (*p* < 0.0004) were significant between the proteinuria and hypertension male group (G3) and male control group (G4). The relative abundances of these structures were subjected to IBM SPSS Statistics to generate box plots. Figure 2 shows the box plot that displays the comparison of the relative abundances of the 19 significant *N*-glycans between these two groups. To validate the significance of these structures, their abundances were also compared using the targeted MRM experiment. Three transitions and collision energy of each *N*-glycans to conduct MRM experiment are listed in Supporting Information Table S3. 

Our results show that high mannose structure 2-9-0-0 was upregulated 2-fold in the male group with proteinuria and hypertension. High mannose chains have been shown to affect binding in the Tamm-Horsfall glycoprotein, the most abundant urinary protein in mammals [89]. Sialylated structures such as 3-4-0-1, and 4-5-0-1 were observed to have overexpression, while 6-6-0-1 was underexpressed in the disease-related group. The alteration of sialylation may affect the functions of sialoglycoproteins, such as podocalyxin in the glomerulus. It has been demonstrated that the loss of interaction between podocalyxin and actin cytoskeleton is related to nephrotic syndromes such as proteinuria [90]. For fucosylated structures, 7-5-1-0, 7-6-1-0, and 7-7-1-0 were downregulated in G3. Sialylated and fucosylated structures such as 6-5-1-1 and 7-5-1-1 were downregulated in G3. Other than these three types of structures, higher abundance was observed with other structures such as 7-8-0-0, while 6-4-0-0, 7-5-0-0, and 7-6-0-0 showed lower in G3 than in G4. Interestingly, among these significant structures, the structures that contained sialic acids were all mono-sialylated structures. In this study, glycan expression in proteinuria and hypertension groups was compared with that in healthy controls. However, in the MWF male model, hypertension and proteinuria began at week 8, escalating to ≥400 mg/24-h urinary albumin excretion at week 32. Therefore, MWF male model (7 weeks) and 32 weeks, which were the best models for our comparison, were selected as healthy control and disease model, respectively. Since the comparison was based on the same gender (male), we realize that the significant expression difference of the glycan might be age-related, or disease related. It has been reported that glycans from 30 *N*-glycosylation sites of the large multiligand endocytic receptor megalin can modulate the ligand-binding capacity [84]. However, due to the small sample size of this study, additional research is required to elucidate conclusive evidence of proteinuria progression that associates with changed protein functions due to the glycan alternations. 

**Figure 3:**
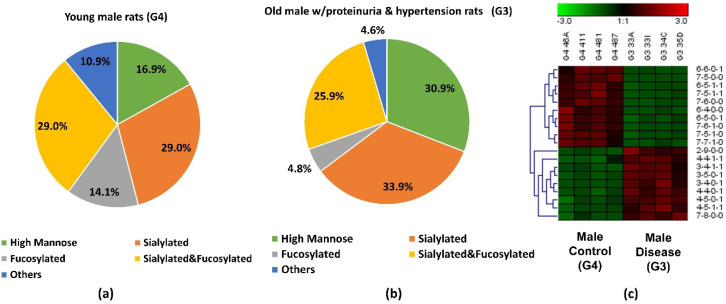
Distribution changes of different *N*-glycan types from (**a**) young male control (G4) and (**b**) old male with proteinuria and hypertension (G3); (**c**) Heatmap of 19 *N*-glycans that exhibited significant expression changes between G3 and G4. In the heatmap, each row represents an individual significant *N*-glycan. The red color of the cell denotes a high relative abundance, while the green color represents a low relative abundance of the *N*-glycan.

The abundance of the 19 significant *N*-glycans took up to 7.3% and 7.2% of the total abundances of glycans in G3 and G4, respectively. When the significant structures were grouped into different types, as shown in Figure 3a,b, the high mannose structure was 30.9% in G3 and 16.9% in G4. In the proteinuria and hypertension male groups, the relative abundance of sialylated structures accounted for 33.9%, while it was 29.0% in male controls. However, the fucosylation level in the diseased-related group (4.8%) was one third of the control group (14.1%). This decrease of fucosylation may have been related to the different expressions of fucosyltransferases in the disease-related and control groups. However, due to the complex enzyme activity that contributed to the *N*-glycosylation modification, the conclusion that different expressions of *N*-glycans are related to specific enzymes is difficult to draw. Since these 19 significant *N*-glycans were low abundance structures, their contribution to the sialylation and fucosylation of the overall *N*-glycans were relatively small. Thus, when comparing overall glycans between these two groups, the sialylation level was increased only 1.7% while the fucosylation level was decreased 0.3% in G3 as shown in Supporting Information Figure S1. For sialylated and fucosylated structures, the overall abundance was observed to decrease in G3 when it compared to G4. Decreased abundance was also obtained in the other structures in G3. Figure 3c depicts the significant expression changes of 19 *N*-glycans between G3 and G4. In this heat map, the four columns on the left represent four replicates from the male control group, while the four columns on the right represent four replicates from the male disease group. Each row represents an individual significant *N*-glycan. The red color of the cell indicates a high relative abundance, while the green color represents a low relative abundance of the *N*-glycan. 10 out of 19 were downregulated in the disease group, and 30% of those were fucosylated structures. For the 9 upregulated expression structures, more than 44% were sialylated. These results were consistent with the results observed in Figure 3a,b.

We also investigated the significant glycan expressions existing in the female proteinuria groups (G1) and the female control group (G2). Here, 6 *N*-glycans were significant (*p* < 0.0004). A comparison of the different relative abundance of these glycans between G1 and G2 appears in Supporting Information Figures S4 and S5. Among the 6 significant glycans, relative abundances of sialylated structures such as 3-6-0-1, 4-6-0-1, and 7-5-0-2 were higher, while 4-4-0-1, and 5-4-0-1 were lower in G1 than in G2. Sialylated and fucosylated structure 3-4-1-1 showed a lower expression in G1 than in G2. The relative abundance of sialylated structures accounted for more than 95% in both groups and a higher sialylation level was observed in the female group with mild proteinuria than the control (Supporting Information Figure S5). In addition to the gender difference, less proteinuria in the female group (G1) than in the male group (G3) may be another factor contributing to the diversity in glycan expression. 

Glycan expression between female and male controls were also compared. A total of 17 *N*-glycans (*p* < 0.0004) were significant in expression between the two healthy controls as shown in Supporting Information Figure S6. Among these significant glycans, the relative abundance of high mannose structures accounted for 23.9% in female controls, while it was 36.2% in male controls. The fucosylation level was 12.2% higher in the female controls than in the male controls. However, the relative abundance of the sialylated and fucosylated structures was 22.7% lower in the female groups than that in the male groups. The observation that significant glycan expressions exist differently between female and male groups was expected, due to the glycosylation features associated with genders that have already been reported [91]. 

### 3.5. Comparison between Obese and Diabetic Group and Control Group

**Figure 4:**
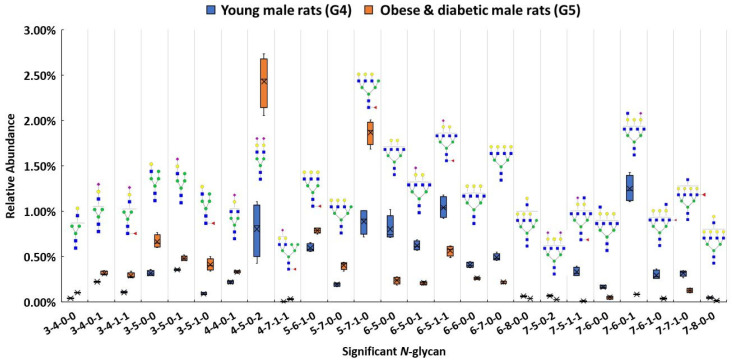
Box plot for relative abundance of 25 significant *N*-glycans (*p* < 0.0004) between young male control (G4) and obese and diabetic male (G5). The putative structure was assigned to each glycan composition. The X axis denotes the four-digit codes for *N*-glycan compositions. The Y axis is the relative abundance. Symbols: see Figure 2.

In addition to investigating glycan expressions in the proteinuria and hypertension group, our study also examined the obese and diabetic group to search for significant glycan expression changes that might be disease related. The use of Zucker diabetic rats was used as a control to show that, as expected, in the glycemic state. The glycans are different than in the other rats which are not diabetic and glycemic. After the Bonferroni correction, a total of 25 *N*-glycans were statistically significant (*p* < 0.0004) between the obese and diabetic male group (G5) and the control (G4). A box plot, as shown in Figure 4, depicts the relative abundance of the 25 significant *N*-glycans between these two groups. Among these significant structures, 7 out of 25 were sialylated, with 4 showing higher relative abundance and 3 showing lower relative abundance in the obese and diabetic group. 

Both 4-4-0-1 and 4-5-0-2 showed higher expressions in G5. In particular, 4-5-0-2 was overexpressed with double the relative abundance in G5 than in G4. On the other hand, 7-6-0-1 was expressed more than four times lower than the relative abundance in G5 as in G4. Significant expressions of these structures were also confirmed with MRM experiment. The significant changes of these *N*-glycan structures may be associated with the obese and diabetic diseases state. Importantly, the alteration of sialylation from the obese and diabetic group is similar to what previous studies have reported [57,91]. For fucosylated structures, upregulated *N*-glycans such as 3-5-1-0 and 5-7-1-0 were expressed in much higher abundance in G5 than in G4. However, 7-6-1-0 and 7-7-1-0 showed lower expression in G5 when compared to G4. Studies have shown that altered fucosylated *N*-glycans were observed in serum samples from Type I diabetic patients, and that inhibition of fucosylation can reduce the progression of diabetic kidney disease [56,92]. The increase of 3-5-1-0 and 5-7-1-0 may play a role in obese and diabetic kidney disease progression. Sialylated and fucosylated structures such as 3-4-1-1 and 4-7-1-1 were upregulated in expression, while 6-5-1-1 and 7-5-1-1 were downregulated in expression in G5 when compared to expression in G4. Other structures such as 3-4-0-0 showed a higher relative abundance in G5, while 6-5-0-0, 6-6-0-0, 6-8-0-0, 7-6-0-0, and 7-8-0-0 displayed lower abundances in G5. Different expressions of all these structures were consistent with the MRM results.

**Figure 5:**
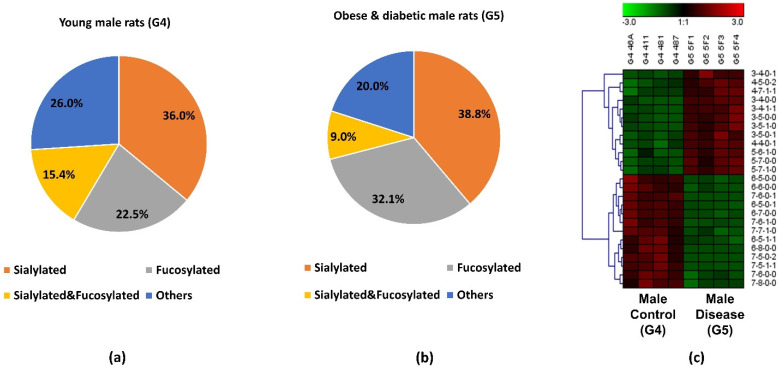
Distribution changes of different *N*-glycan types from (**a**) young male control (G4) and (**b**) obese and diabetic male (G5); (**c**) Heatmap of 25 *N*-glycans that exhibited significant expression changes between G4 and G5. In the heatmap, each row represents an individual significant *N*-glycan. The red color of the cell denotes a high relative abundance, while the green color represents a low relative abundance of the *N*-glycan.

The pie charts shown in Figure 5a,b depict the distribution changes of different *N*-glycan types of the 25 significant *N*-glycans from G5 and G4. The abundance of these structures took up to 9.8% and 10.1% of the total abundances of glycans in G4 and G5, respectively. Figure 5a shows the distribution in the G4 control. Sialylated structures were the most abundant and took up 36.0%; 22.5% of the structures were fucosylated; and 15.4% were sialylated and fucosylated. Other structures without sialic acids and fucoses accounted for 26.0% of the total abundances. Compared to the distribution of sialylated structures from the control group, a higher sialylation level was observed in G5, of which sialylated glycans took up to 38.8%. As shown in Supporting Information Figure S1, an increase sialylation level (1.6%) was observed in the overall glycan expression in the G5. Because these 25 significant structures only took up approximately 10% of the overall abundance, their contribution to the overall sialylation level was relatively low. Moreover, 32.1% was fucosylated, which was 9.6% higher than in G4. However, the sialylated and fucosylated structures were 6.4% lower, and other structures were 6% lower than in G4. Figure 5c displays the heatmap of differentially expressed *N*-glycans between G4 and G5. Each row represents an individual glycan that was statistically significant between these two groups (*p* < 0.0004 with Bonferroni correction). Among these 25 significant *N*-glycans, 12 of them were upregulated in G5, with 4 sialylated, 3 fucosylated, 2 sialylated and fucosylated, and 3 others. The remaining 13 had lower abundance in G5, with 3 sialylated, 2 fucosylated, 2 sialylated and fucosylated, and 6 others. The increased abundance of sialylated *N*-glycans and fucosylated *N*-glycans may be important to the progression of obese and diabetic diseases, which should be further explored in future research. Zucker diabetic rats were used as a control to show that, as expected, in the glycemic state the glycans are different than in the other rats (MWF rats at 7 weeks age) which are not diabetic and glycemic. Since these two rat models were from two different strains, the limitation is that the significant expression of the glycan might be strain-, age- and disease-related. Our objective was to address whether significant glycan changes could be observed in the BBM from an established rat kidney disease model not having hyperglycemia. The MWF rat offer a unique opportunity to follow physiological changes as they age while also enabling direct visualization of glomerular filtration and Proximal tubule cell uptake at the BBM since they contain surface glomeruli. Characterizing the glycan changes in young male (G4) versus old male that have proteinuria and hypertension (G3) was the primary focus of this study.

## 4. Conclusions

In this work, kidney BBMs from rats, which had different physiological states and levels of proteinuria, had their *N*-glycomics profiles investigated and quantitatively characterized. Several techniques were combined to accomplish this, including BBM purification, *N*-glycan purification, reduction, permethylation, and finally efficient and sensitive Nano-LC-MS analysis. There are 121 glycans structures that were identified and quantified so the relative abundance to each individual structure was relatively low in most cases. Although their relative abundances were low, their S/N ratio were much higher than the limit of quantitation (S/N = 10). In addition, their signal intensities were higher than 1.00 × 10^5^. Take the lowest abundance structures (HexNAc_7_Hex_6_DeoxyHex_1_) with relative abundance less than 0.05% as an example, the signal intensity of this structure was 4.33 × 10^5^ ± 1.42 × 10^5^. The S/N was 2193.25 ± 69.288 which is about 210 times higher than the limit of quantitation. In this case, LC-MS/MS can detect all these low abundant structures and provided reliable data for quantification. 

*N*-glycomics profiles of both control groups and three kidney disease groups including aged with mild proteinuria (G1), aged with proteinuria and hypertension (G3), obesity and diabetes (G5) were determined. To avoid any effect of gender, the comparison was accomplished within same-gender groups. Both female groups with mild proteinuria (G1) and male groups with severe proteinuria and hypertension (G3) exhibited distinct *N*-glycan expressions compared to two control groups. 6 *N*-glycans showed significant expressions in G1, and five of them were sialylated structures. The sialylation level was higher in G3, indicating that glycan sialylation may be associated with proteinuria and hypertension progress. The alteration of sialylation may affect the functions of sialoglycoproteins, such as podocalyxin in the glomerulus, which is associated with nephrotic syndromes such as proteinuria. Other than the sialylated structures, the high mannose structure 2-9-0-0 was upregulated 2-fold in G3. The changed expression of the high mannose structure may impact the binding of the Tamm-Horsfall glycoprotein, which is related to the progression of proteinuria. The overexpression of 4-5-0-2 and the lower expression of 7-6-0-1 were observed in G5, suggesting that they may play an important role in obese and diabetic progress. In addition, fucosylated glycans such as 3-5-1-0 and 5-7-1-0 were expressed in much higher abundance in G5, signifying that they may be essential indicators in the progress of obese and diabetic disease. The distinct glycan expressions revealed through this glycomics analysis provide an important first step in defining relevant BBM glycan changes occurring with disease and determining how they may impact function while also leading to discovery of potential glycan biomarkers to improve therapy for CKD patients. 

## Figures and Tables

**Table 1:** Different specifications of five groups of rats.

Group	G1	G2	G3	G4	G5
Rat Strain	Munich Wistar Frömter (MWF)	MWF	MWF	MWF	Zucker SF1 (ZSF1) obese
Gender	Female	Female	Male	Male	Male
Age–weeks	32–42	<10	32–42	7	16
Physiology	Mild proteinuria	Normal	Proteinuria & mild hypertension	Normal	Obese, hypertension, hyperlipidemia, hyperglycemia
Urinary protein (mg/day)	<100	Negligible	>400	<50	~400

## Data Availability

Research data used in this article are available from the corresponding author on request.

## Supplementary Materials

The following are available online at https://www.mdpi.com/article/10.3390/biom11111677/s1, Figure S1: Distribution of the different types of *N*-glycans among five groups, Figure S2: An example of *N*-glycan identification with full MS and MS^2^, Figure S3: The PCA plots for (a) the old female group with proteinuria (G1) versus the young female control group (G2), and (b) the young female control group (G2) versus the young male control group (G4), Figure S4: Box plot for relative abundance of significant *N*-glycans (*p* < 0.0004) between the young female control group (G2) and the old female group with proteinuria (G1), Figure S5: Distribution of the types of *N*-glycans and a heatmap of significant *N*-glycans from the young female control group (G2) and the old female group with proteinuria (G1), Figure S6: Box plot for relative abundance of significant *N*-glycans (*p* < 0.0004) between the female control group (G2) and the male control group (G4). Figure S7: Distribution of the types of *N*-glycans and a heatmap of significant *N*-glycans from the young female control group (G2) and the young male control group (G4). Table S1: List of all identified *N*-glycans with their theoretical m/z, observed m/z, average relative abundance, standard deviation, and *p*-value for each sample group, Table S2: This table is presented in an attached Excel file which contains a list of statistically significant *N*-glycans (*p* < 0.0004) between different groups. Table S3: Transitions used for the quantitation of permethylated *N*-glycans detected in five BBM groups for MRM LC-MS/MS.
