# Supplementary material for: Changes in the Expression of Renal Brush Border Membrane N-Glycome in Model Rats with Chronic Kidney Diseases
Source: Biomolecules. 2021 Nov 11;11(11):1677. doi: 10.3390/biom11111677 (PMC8616023; doi:10.3390/biom11111677)
Supplement: Supplementary file 1 [file biomolecules-11-01677-s001.zip › biomolecules-1399453-supplementary.pdf]

Supporting Information

Changes in the Expression of Renal Brush Border Membrane N-Glycome in Model Rats with Chronic Kidney Diseases

Aiying Yu[1§], Jingfu Zhao[1§], Shiv Pratap S. Yadav[2], Bruce A Molitoris[2], Mark C Wagner[2], and Yehia Mechref[*,1]

[1]Department of Chemistry and Biochemistry, Texas Tech University, Texas, TX, 79409, United States; aiying.yu@ttu.edu (A.Y.); Jingfu.zhao@ttu.edu (J.Z.).

[2]Nephrology Division, Department of Medicine, Indiana University, Indianapolis, IN, 46202, United States; ssyadav@iu.edu (S.P.S.Y.); bmolitor@iu.edu (B.A.M.); wagnerm@iu.edu (M.C.W.).

*Correspondence: yehia.mechref@ttu.edu (Y.M.); Tel: 806-742-3059

[§]These authors contributed equally to this work.

Keywords: Glycomic, Brush-border membrane, Chronic kidney disease, Proteinuria and hypertension, Obese and diabetic, LC-MS/MS

Table of Contents

Supporting Information Table S1. List of all identified *N*-glycans with their theoretical m/z, observed m/z, average relative abundance, standard deviation, and *p*-value for each sample group.

Supporting Information Table S2. This table is presented in an attached Excel file which contains a list of statistically significant *N*-glycans (p<0.0004) between old male group with proteinuria and hypertension (G3) *versus* young male control group (G4), Obese and diabetic male group (G5) *versus* young male control group (G4), old female group with proteinuria (G1) *versus* young female control group (G2), and young female control group (G2) *versus* young male control group (G4) and their relative abundance with *p*-value.

Supporting Information Table S3. Transitions used for the quantitation of permethylated *N*-glycans detected in five BBM groups for MRM LC-MS/MS.

Supporting Information Figure S1. Distribution of the different types of *N*-glycans among five groups.

Supporting Information Figure S2. An example of *N*-glycan (HexNAc$_4$Hex$_5$DeoxyHex$_1$NeuAc$_1$) identification with full MS and MS^2.

Supporting Information Figure S3. The PCA plots for **(a)** the old female group with proteinuria (G1) *versus* the young female control group (G2), and **(b)** the young female control group (G2) *versus* the young male control group (G4).

Supporting Information Figure S4. Box plot for relative abundance of significant *N*-glycans (p<0.0004) between the young female control group (G2) and the old female group with proteinuria (G1).

Supporting Information Figure S5. Distribution of the types of *N*-glycans and a heatmap of significant *N*-glycans from the young female control group (G2) and the old female group with proteinuria (G1).

Supporting Information Figure S6. Box plot for relative abundance of significant *N*-glycans (p<0.0004) between the young female control group (G2) and the young male control group (G4).

Supporting Information Figure S7. Distribution of the types of *N*-glycans and a heatmap of significant *N*-glycans from the young female control group (G2) and the young male control group (G4).

Table S3. Transitions used for the quantitation of permethylated *N*-glycans detected in five BBM groups for MRM LC-MS/MS. Symbols: see **Figure S2.**

Structures	*m/z* (charge)	Transitions			Collision Energy
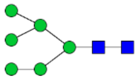	897.983 (+2)	${}^{0,2}X$ 111	$BZ^{0,2}X$ 230	$B^{0,2}X$ 262	35
* 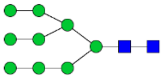	1204.133 (+2)	BZ 187	$B^{0,2}X$ 262	$C^{2,5}X$ 294	35
# 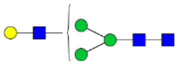	807.934 (+2)	BZZ 196	BZ 228	B 464	30
* 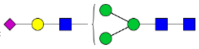	988.520 (+2)	BZ 344	B 376	B 825	30
# 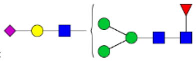	1075.565 (+2)	BZ 344	B 376	B 825	30
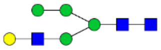	909.983 (+2)	BZZ 196	BZ 228	B 260	30
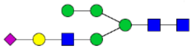	1090.570 (+2)	BZ 344	B 376	B 825	30
# 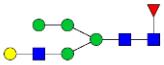	997.028 (+2)	BZZ 196	BZ 228	B 260	35
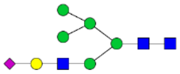	1192.620 (+2)	BZ 344	B 376	B 825	30
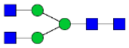	828.446 (+2)	BZZ 196	BZ 228	B 260	30
# 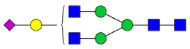	1111.083 (+2)	BZ 344	B 376	B 825	30
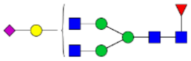	1198.128 (+2)	BZZ 312	BZ 344	B 376	30
* 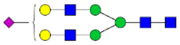	1213.133 (+2)	BZ 344	B 376	B 825	30

# 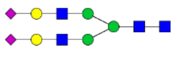	929.482 (+3)	BZ 344	B 376	B 825	30
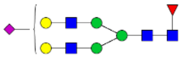	1300.177 (+2)	BZZ 312	BZ 344	B 376	30
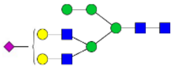	1315.183 (+2)	BZ 344	B 376	B 825	30
# 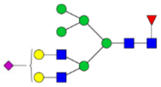	1003.187 (+3)	BZ 344	B 376	B 825	30
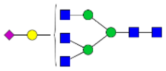	1233.646 (+2)	BZ 344	B 376	B 825	30
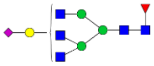	1320.691 (+2)	BZZ 312	BZ 344	B 376	30
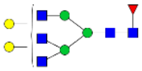	1242.154 (+2)	BZZ 196	B 260	B 464	35
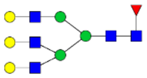	896.472 (+3)	BZZ 196	BZ 228	B 464	35
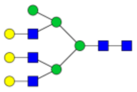	906.475 (+3)	BZZ 196	B 260	B 464	35
# 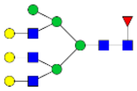	964.505 (+3)	BZZ 196	B 260	B 464	35
* 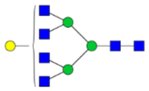	1175.622 (+2)	BZZ 196	BZ 228	B 260	35
# 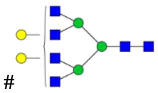	1277.672 (+2)	BZZ 196	B 260	B 464	30
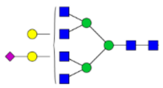	1458.259 (+2)	BZZ 312	BZ 344	B 376	30
*# 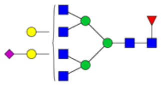	1030.538 (+3)	BZZ 312	BZ 344	B 376	30
# 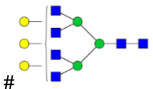	920.151 (+3)	BZZ 196	B 260	B 464	30

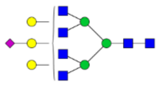 *	1040.542 (+3)	BZZ 312	BZ 344	B 376	30
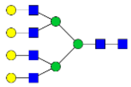	988.184 (+3)	BZZ 196	B 260	B 464	30
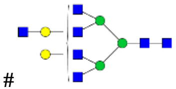 #	1056.218 (+3)	BZ 228	B 260	B 464	35
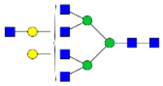 *	933.826 (+3)	BZ 228	B 260	B 464	35
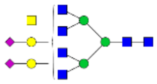	1174.609 (+3)	BZ 344	B 376	B 825	30
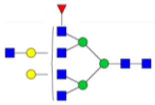 *	991.856 (+3)	BZZ 196	B 260	B 464	35
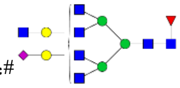 *#	1112.247 (+3)	BZZ 312	BZ 344	B 376	30
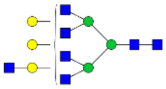 *#	1001.859 (+3)	BZ 228	B 260	B 464	35
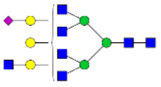 #	1122.251 (+3)	BZ 344	B 376	B 825	30
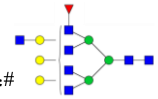 *#	1059.889 (+3)	BZ 228	B 260	B 464	35
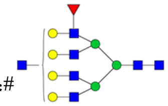 *#	1127.923 (+3)	BZ 228	B 260	B 464	35
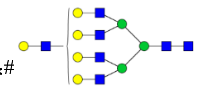 *#	1137.926 (+3)	BZZ 196	BZ 228	B 464	35
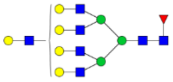	1195.956 (+3)	BZZ 196	BZ 228	B 464	35

*Significant *N*-glycans ($p<0.05$) between old male group with proteinuria and hypertension (G3) and young male control group (G4) were confirmed from MRM experiment.

#Significant *N*-glycans ($p<0.05$) between obese and diabetic male group (G5) and young male control group (G4) were confirmed from MRM experiment.

Supporting Information Figure S1

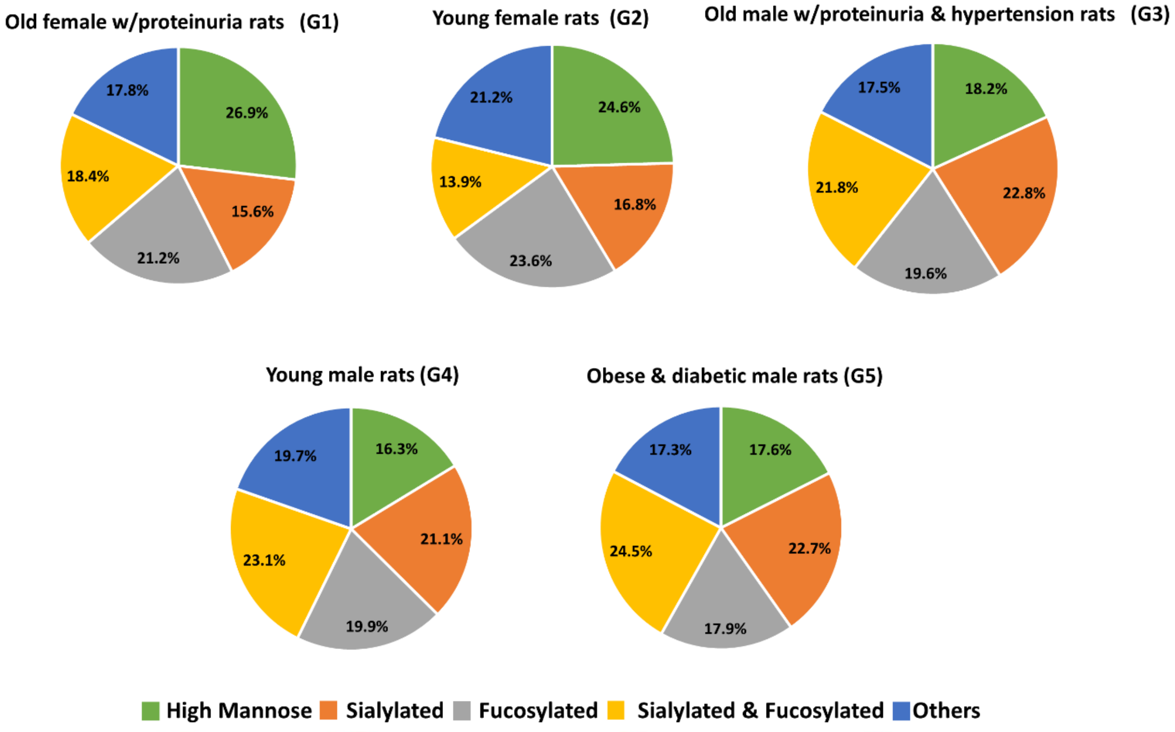

Supporting Information Figure S1. Distribution of the different *N*-glycans types among five groups.

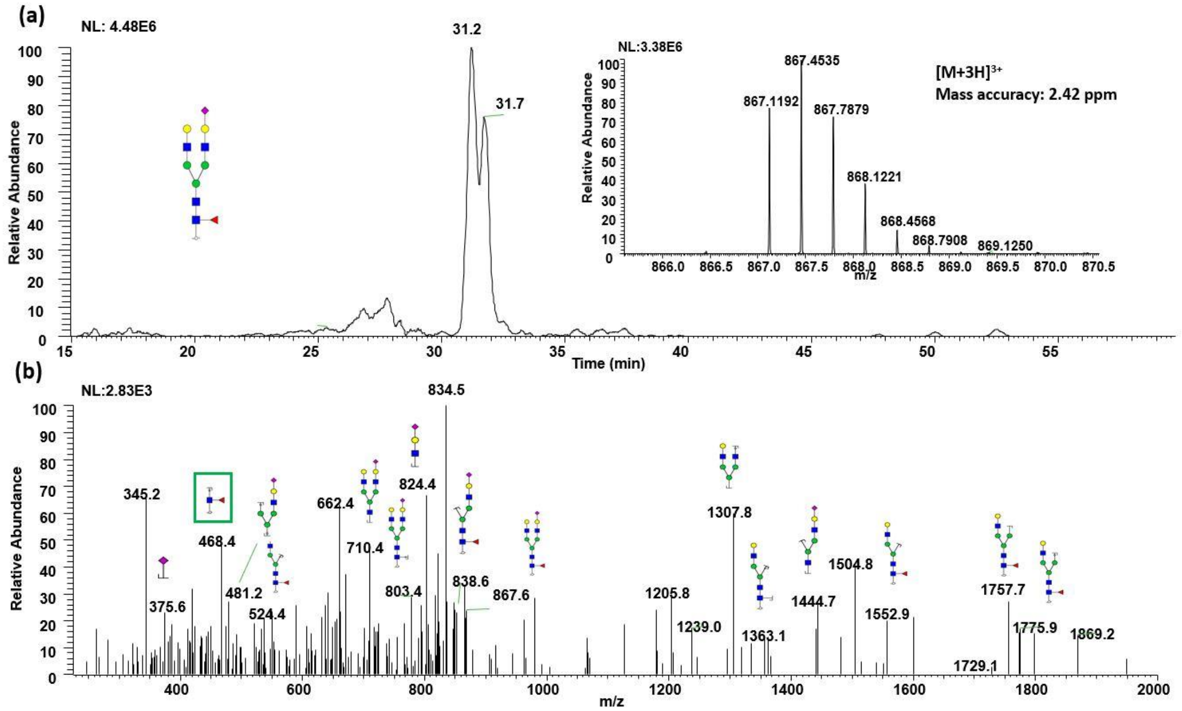

Supporting Information Figure S2. An example of *N*-glycan identification with full MS and MS^2. **(a)** The EIC of *N*-glycan 4-5-1-1 ($HexNAc_4Hex_5DeoxyHex_1NeuAc_1$) at retention time 31.2 and 31.7 min, inset is the full MS. **(b)** MS^2 for $HexNAc_4Hex_5DeoxyHex_1NeuAc_1$, where the fragment with a green frame is the diagnostic ion for core fucose. HexNAc includes *N*-acetylglucosamine and N-acetylgalactosamine. Hexose includes galactose and mannose. Deoxyhexose is fucose and NeuAc is *N*-acetylneuraminic acid. Symbols: ■, *N*-acetylglucosamine (GlcNAc); ○ , Galactose (Gal); ▼ , Fucose (Fuc); ●, Mannose (Man); ●, Glucose (Glc); ◆, *N*-acetylneuraminic acid (NeuAc/Sialic Acid).

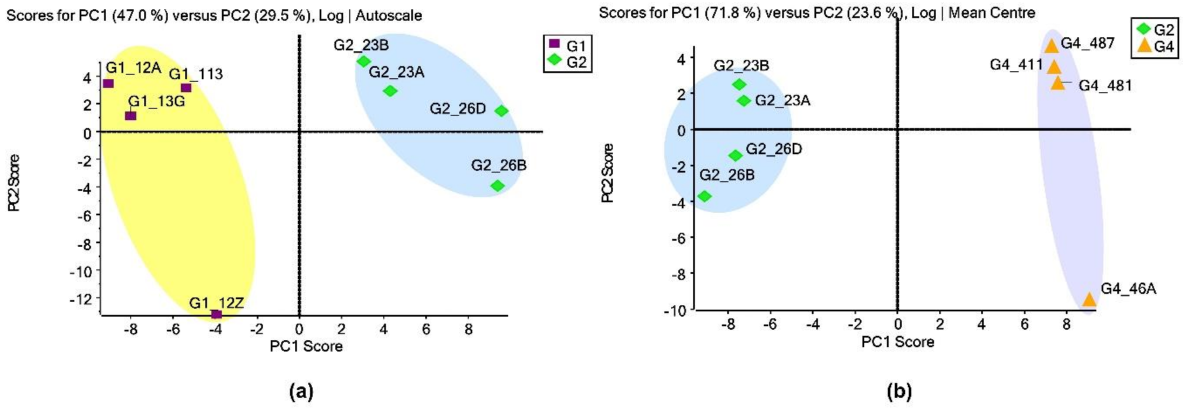

Supporting Information Figure S3. The PCA plots for **(a)** the old female group with proteinuria (G1) *versus* the young female control group (G2), and **(b)** the young female control group (G2) *versus* the young male control group (G4).

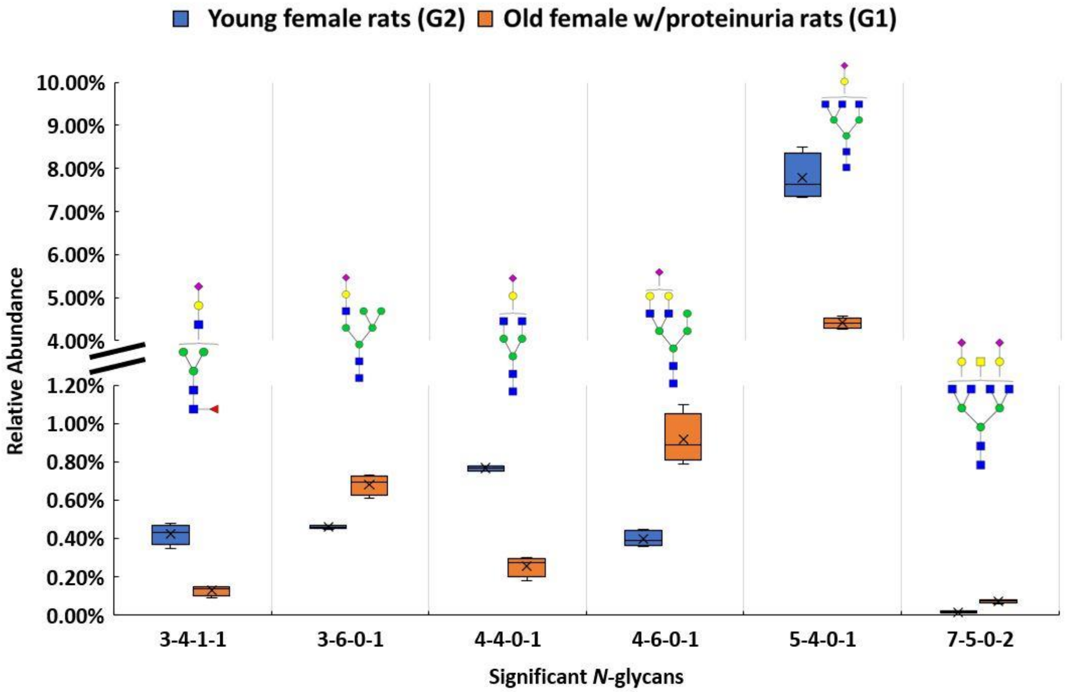

Supporting Information Figure S4. Box plot for relative abundance of significant *N*-glycans ($p<0.0004$) between the young female control group (G2) and the old female group with mild proteinuria (G1). The putative structure was assigned to each glycan composition. The X axis denotes the four-digit codes for *N*-glycan compositions. The Y axis is the relative abundance. The four-digit codes represent *N*-glycan compositions. X-X-X-X stands for HexNAc-Hexose-DeoxyHex-NeuAc. Symbols: see **Figure S2**.

Supporting Information Figure S5

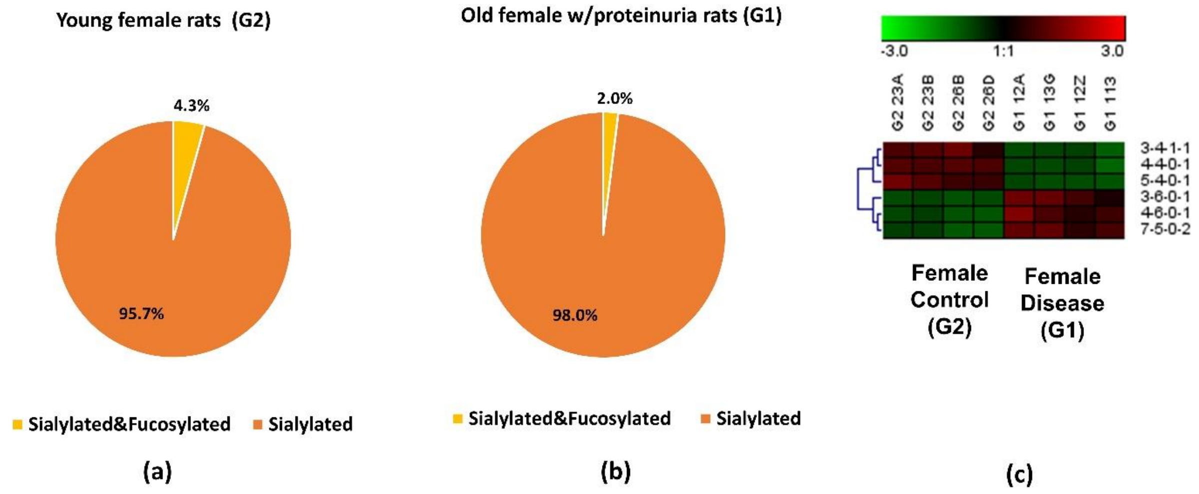

Supporting Information Figure S5. Distribution of the types of *N*-glycans derived from **(a)** the young female control group (G2) and the **(b)** old female group with mild proteinuria (G1); **(c)** Heatmap of 6 *N*-glycans that exhibited significant expression changes between G2 and G1. In the heatmap, each row represents an individual significant *N*-glycan. The red color of the cell denotes a high relative abundance, while the green color represents a low relative abundance of the *N*-glycan.

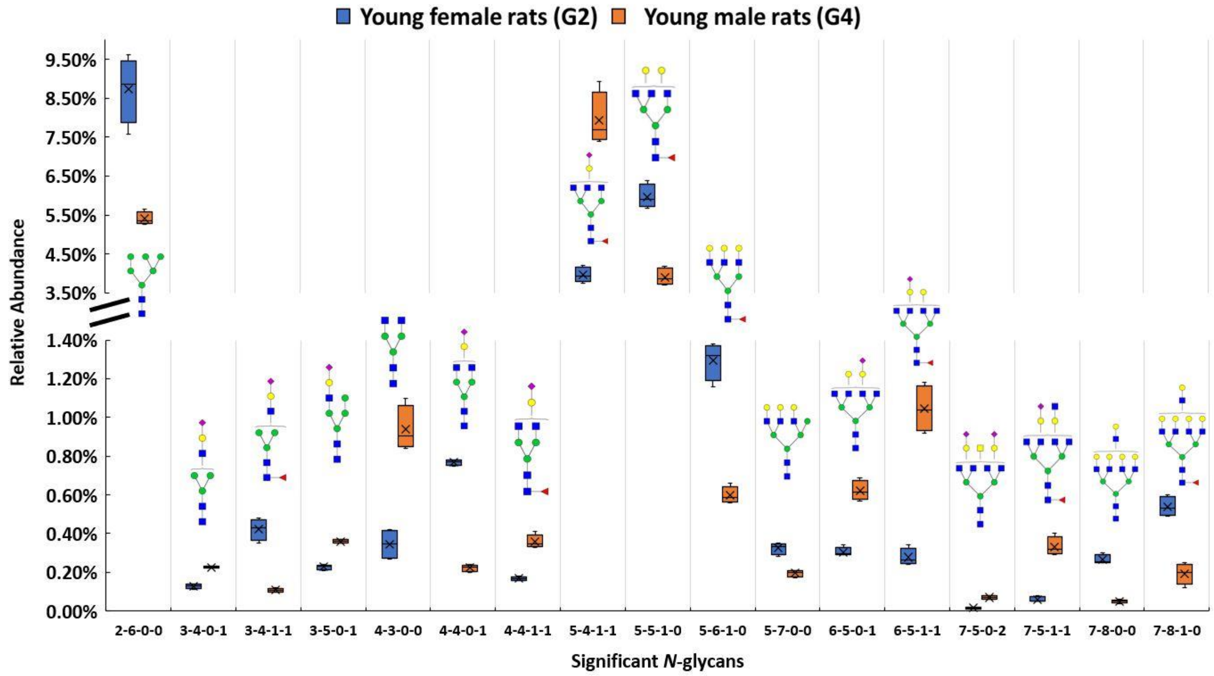

Supporting Information Figure S6. Box plot for relative abundance of significant *N*-glycans ($p<0.0004$) between the young female control group (G2) and the young male control group (G4). The putative structure was assigned to each glycan composition. The X axis denotes the four-digit codes for *N*-glycan compositions. The Y axis is the relative abundance. Symbols: see **Figure S2**.

Supporting Information Figure S7

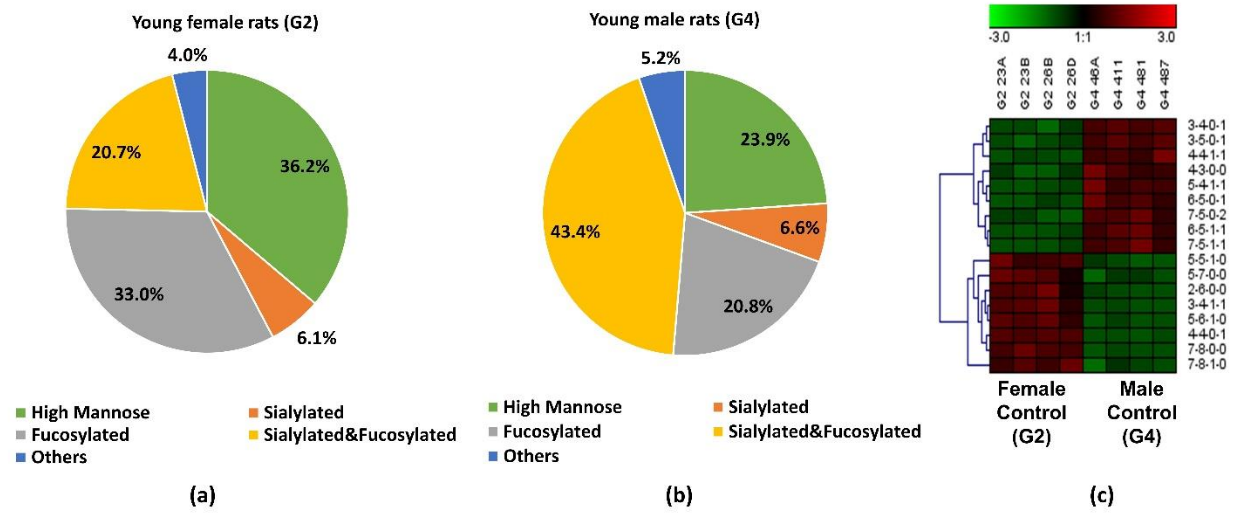

Supporting Information Figure S7. Distribution of the types of *N*-glycans derived from **(a)** the female control group (G2) and **(b)** the male control group (G4); **(c)** Heatmap of 17 *N*-glycans that exhibited significant expression changes between G2 and G4. In the heatmap, each row represents an individual significant *N*-glycan. The red color of the cell denotes a high relative abundance, while the green color represents a low relative abundance of the *N*-glycan.

Table S1. Relative abundance and putative *N*-glycan structures of Brush Border Membrane protein samples using LC-MS/MS. Symbols: , N-acetylglucosamine (GlcNAc); , Galactose (Gal); , Fucose (Fuc); , Mannose (Man); , N-acetylneuraminic acid (NeuAc/Sialic Acid)

p-value

Composition: X-X-X-X stands for HexNAc-Hexose-DeoxyHex-NeuAc. HexNAc includes N-acetylglucosamine and N-acetylgalactosamine. Hexose includes galactose and mannose. Deoxyhexose is fucose and NeuAc is N-acetylneuraminic acid. G1: old female group with proteinuria; G2: young female group; G3: old male group with proteinuria and hypertension; G4: young male group; G5: male group with obesity and diabetics.

Glycan	Composition	Theoretical m/z	Observed m/z	Mass accuracy (ppm)	G1 12A	G1 13G	G1 12Z	G1 113	Average R	Standard I	G2 23A	G2 23B	G2 26B	G2 26D	Average R	Standard I	G3 33A	G3 33I	G3 34C	G3 35D	Average R	Standard I	G4 46A	G4 411	G4 481	G4 487	Average R	Standard I	G5 5F1	G5 5F2	G5 5F3	G5 5F4	Average R	Standard I	G2 vs G1	G3 vs G1	G4 vs G1	G5 vs G1	G3 vs G2	G4 vs G2	G5 vs G2	G4 vs G3	G5 vs G3	G5 vs G4
	2-5-0-0	787.4203	787.4187	2.03	4.99E-02	6.28E-02	7.13E-02	4.87E-02	5.82E-02	1.08E-02	5.51E-02	4.70E-02	5.17E-02	5.11E-02	5.12E-02	3.32E-03	7.14E-02	5.62E-02	8.59E-02	5.84E-02	6.80E-02	1.37E-02	6.38E-02	6.01E-02	5.68E-02	5.30E-02	5.84E-02	4.61E-03	7.13E-02	7.46E-02	8.11E-02	7.73E-02	7.61E-02	4.14E-03	0.3	0.3	1	0.02	0.05	0.04	0.00008	0.2	0.3	0.001
	2-6-0-0	889.4702	889.4690	1.35	7.53E-02	8.58E-02	9.74E-02	9.05E-02	8.73E-02	9.28E-03	8.80E-02	8.94E-02	9.63E-02	7.58E-02	8.74E-02	8.52E-03	5.65E-02	4.22E-02	4.90E-02	5.01E-02	4.95E-02	5.89E-03	5.64E-02	5.39E-02	5.27E-02	5.30E-02	5.40E-02	1.70E-03	5.07E-02	4.73E-02	4.98E-02	4.70E-02	4.87E-02	1.83E-03	1	0.0005	0.0004	0.0002	0.0003	0.0003	0.0001	0.2	0.8	0.005
	2-7-0-0	991.5201	991.5181	2.02	5.05E-02	5.66E-02	7.17E-02	7.18E-02	6.27E-02	1.08E-02	5.96E-02	6.13E-02	6.60E-02	4.53E-02	5.80E-02	8.94E-03	2.48E-02	1.70E-02	2.30E-02	2.41E-02	2.22E-02	3.54E-03	2.90E-02	2.80E-02	2.80E-02	2.39E-02	2.72E-02	2.28E-03	2.38E-02	2.16E-02	2.27E-02	2.14E-02	2.24E-02	1.12E-03	0.5	0.0004	0.0007	0.0003	0.0003	0.0005	0.0002	0.06	0.9	0.009
	2-8-0-0	1093.5700	1093.5678	2.01	2.06E-02	2.21E-02	3.18E-02	2.95E-02	2.60E-02	5.48E-03	1.88E-02	2.33E-02	2.71E-02	1.63E-02	2.14E-02	4.81E-03	1.82E-02	1.19E-02	1.24E-02	1.29E-02	1.39E-02	2.95E-03	9.53E-03	6.25E-03	5.68E-03	6.44E-03	6.97E-03	1.74E-03	9.82E-03	7.17E-03	7.95E-03	6.65E-03	7.90E-03	1.39E-03	0.3	0.008	0.0006	0.0007	0.04	0.001	0.002	0.007	0.01	0.4
	2-9-0-0	1195.6199	1195.6182	1.42	2.62E-02	2.63E-02	3.42E-02	2.70E-02	2.84E-02	3.84E-03	2.00E-02	2.38E-02	2.74E-02	1.93E-02	2.26E-02	3.76E-03	2.71E-02	2.20E-02	2.10E-02	2.20E-02	2.30E-02	2.77E-03	1.22E-02	1.31E-02	1.21E-02	1.15E-02	1.22E-02	6.53E-04	1.56E-02	1.41E-02	1.57E-02	1.36E-02	1.47E-02	1.04E-03	0.08	0.06	0.0002	0.0005	0.9	0.002	0.007	0.0003	0.001	0.006
	2-10-0-0	1297.6698	1297.6694	0.31	6.68E-03	5.51E-03	7.25E-03	6.20E-03	6.41E-03	7.37E-04	5.04E-03	4.75E-03	5.31E-03	5.00E-03	5.02E-03	2.28E-04	5.50E-03	5.34E-03	5.23E-03	5.49E-03	5.39E-03	1.31E-04	3.04E-03	5.36E-03	4.95E-03	3.84E-03	4.30E-03	1.05E-03	5.07E-03	5.39E-03	6.22E-03	6.50E-03	5.79E-03	6.75E-04	0.01	0.03	0.02	0.3	0.03	0.2	0.07	0.09	0.3	0.05
	3-3-0-0	705.8837	705.8821	2.20	1.28E-03	1.98E-03	1.60E-03	1.37E-03	1.56E-03	3.13E-04	1.50E-03	1.27E-03	7.77E-04	1.44E-03	1.25E-03	3.29E-04	1.89E-03	1.79E-03	2.77E-03	1.90E-03	2.09E-03	4.59E-04	1.43E-03	1.48E-03	1.40E-03	1.45E-03	1.44E-03	3.26E-05	1.44E-03	2.07E-03	1.54E-03	1.85E-03	1.73E-03	2.87E-04	0.2	0.1	0.5	0.5	0.02	0.3	0.07	0.03	0.2	0.09
	3-3-1-0	792.9283	792.9265	2.21	8.40E-04	1.12E-03	1.11E-03	7.87E-04	9.65E-04	1.77E-04	1.20E-03	7.41E-04	5.62E-04	8.43E-04	8.36E-04	2.68E-04	2.01E-03	1.95E-03	3.60E-03	1.91E-03	2.37E-03	8.21E-04	2.51E-03	2.40E-03	2.01E-03	1.73E-03	2.16E-03	3.61E-04	2.77E-03	3.12E-03	2.90E-03	3.39E-03	3.05E-03	2.70E-04	0.5	0.02	0.001	0.00001	0.01	0.001	0.00002	0.7	0.2	0.008
	3-4-0-0	807.9336	807.9315	2.54	2.17E-04	3.34E-04	3.04E-04	1.96E-04	2.63E-04	6.65E-05	4.95E-04	3.92E-04	4.33E-04	5.32E-04	4.63E-04	6.28E-05	7.18E-04	6.83E-04	1.02E-03	6.69E-04	7.72E-04	1.65E-04	5.18E-04	4.11E-04	3.94E-04	3.73E-04	4.24E-04	6.44E-05	9.53E-04	1.00E-03	1.07E-03	1.14E-03	1.04E-03	8.21E-05	0.005	0.001	0.01	6E-06	0.01	0.4	0.00003	0.008	0.03	0.00002
	3-4-0-1	988.5204	988.5184	2.02	1.82E-03	1.85E-03	2.22E-03	1.34E-03	1.81E-03	3.61E-04	1.31E-03	1.29E-03	1.14E-03	1.36E-03	1.27E-03	9.54E-05	5.33E-03	4.66E-03	5.82E-03	4.37E-03	5.04E-03	6.54E-04	2.25E-03	2.30E-03	2.21E-03	2.25E-03	2.25E-03	3.77E-05	3.02E-03	3.46E-03	3.12E-03	3.11E-03	3.18E-03	1.94E-04	0.03	0.0001	0.05	0.0005	0.00003	1E-06	2E-06	0.0001	0.002	0.00008
	3-4-1-0	894.9782	894.9753	3.18	2.01E-04	2.23E-04	2.55E-04	1.46E-04	2.06E-04	4.56E-05	1.92E-04	1.36E-04	9.11E-05	1.38E-04	1.39E-04	4.12E-05	3.84E-04	3.12E-04	5.88E-04	3.11E-04	3.99E-04	1.30E-04	1.82E-04	2.24E-03	3.04E-04	2.69E-04	7.50E-04	9.98E-04	2.23E-04	3.73E-04	2.69E-04	2.39E-04	2.76E-04	6.76E-05	0.07	0.03	0.3	0.1	0.009	0.3	0.01	0.5	0.1	0.4
	3-4-1-1	1075.5650	1075.5606	4.09	1.32E-03	1.36E-03	1.49E-03	9.46E-04	1.28E-03	2.33E-04	4.17E-03	4.40E-03	4.80E-03	3.53E-03	4.23E-03	5.29E-04	2.73E-03	2.45E-03	2.64E-03	2.02E-03	2.46E-03	3.16E-04	1.12E-03	1.23E-03	9.73E-04	1.05E-03	1.09E-03	1.10E-04	2.66E-03	2.78E-03	2.77E-03	3.37E-03	2.90E-03	3.18E-04	0.00005	0.001	0.2	0.0002	0.001	0.00002	0.005	0.0002	0.1	0.00004
	3-5-0-0	909.9835	909.9810	2.69	2.91E-03	3.38E-03	4.42E-03	2.50E-03	3.31E-03	8.28E-04	2.55E-03	2.17E-03	2.53E-03	2.71E-03	2.49E-03	2.31E-04	3.98E-03	3.23E-03	4.10E-03	3.37E-03	3.67E-03	4.33E-04	3.61E-03	3.24E-03	2.93E-03	3.02E-03	3.20E-03	3.03E-04	6.26E-03	6.03E-03	6.73E-03	7.66E-03	6.67E-03	7.22E-04	0.1	0.5	0.8	0.0009	0.003	0.01	0.00003	0.1	0.0004	0.0001
	3-5-0-1	1090.5703	1090.5682	1.93	3.40E-03	3.36E-03	3.56E-03	2.82E-03	3.28E-03	3.21E-04	2.33E-03	2.13E-03	2.32E-03	2.37E-03	2.29E-03	1.06E-04	5.44E-03	5.27E-03	5.43E-03	4.84E-03	5.24E-03	2.83E-04	3.53E-03	3.71E-03	3.47E-03	3.69E-03	3.60E-03	1.21E-04	4.75E-03	4.63E-03	5.20E-03	4.73E-03	4.83E-03	2.52E-04	0.001	0.0001	0.1	0.0003	1E-06	3E-06	2E-06	0.00004	0.07	0.0001
	3-5-1-0	997.0281	997.0253	2.76	1.41E-03	1.38E-03	1.88E-03	9.55E-04	1.41E-03	3.78E-04	1.00E-03	8.15E-04	8.65E-04	8.69E-04	8.88E-04	8.12E-05	1.53E-03	1.32E-03	1.79E-03	1.25E-03	1.47E-03	2.40E-04	1.06E-03	9.61E-04	8.05E-04	8.18E-04	9.11E-04	1.22E-04	3.89E-03	3.36E-03	4.18E-03	5.04E-03	4.12E-03	6.99E-04	0.04	0.8	0.05	0.0005	0.004	0.8	0.00009	0.006	0.0004	0.0001
	3-5-1-1	1177.6149	1177.6125	2.04	1.48E-03	1.60E-03	1.52E-03	1.42E-03	1.50E-03	7.68E-05	1.34E-03	1.24E-03	9.11E-04	9.39E-04	1.11E-03	2.15E-04	1.57E-03	1.72E-03	2.06E-03	1.49E-03	1.71E-03	2.52E-04	1.40E-03	1.45E-03	1.26E-03	1.09E-03	1.30E-03	1.59E-04	1.46E-03	1.45E-03	1.61E-03	1.44E-03	1.49E-03	7.88E-05	0.01	0.2	0.06	0.8	0.01	0.2	0.02	0.03	0.1	0.08
	3-6-0-0	1012.0334	1012.0311	2.22	1.88E-03	1.94E-03	2.72E-03	1.31E-03	1.96E-03	5.79E-04	1.28E-03	1.18E-03	1.56E-03	1.43E-03	1.36E-03	1.66E-04	2.72E-03	2.09E-03	2.44E-03	2.06E-03	2.33E-03	3.14E-04	1.33E-03	1.30E-03	1.20E-03	1.35E-03	1.30E-03	6.52E-05	1.96E-03	1.46E-03	1.89E-03	1.93E-03	1.81E-03	2.37E-04	0.09	0.3	0.06	0.6	0.002	0.5	0.02	0.0007	0.04	0.006
	3-6-0-1	1192.6202	1192.6164	3.19	7.25E-03	7.24E-03	6.75E-03	6.08E-03	6.83E-03	5.56E-04	4.63E-03	4.72E-03	4.55E-03	4.59E-03	4.62E-03	7.35E-05	8.95E-03	9.50E-03	9.44E-03	9.21E-03	9.27E-03	2.51E-04	4.71E-03	7.03E-03	6.41E-03	6.58E-03	6.18E-03	1.02E-03	9.70E-03	9.22E-03	1.10E-02	9.76E-03	9.92E-03	7.52E-04	0.0002	0.0002	0.3	0.0006	0	0.02	8E-06	0.001	0.2	0.001
	3-6-1-1	1279.6648	1279.6613	2.74	1.60E-03	1.45E-03	1.21E-03	1.11E-03	1.35E-03	2.24E-04	7.68E-04	7.97E-04	6.82E-04	7.18E-04	7.41E-04	5.14E-05	2.33E-03	1.92E-03	2.01E-03	2.21E-03	2.12E-03	1.83E-04	1.28E-03	1.98E-03	1.68E-03	1.73E-03	1.67E-03	2.91E-04	1.08E-03	1.18E-03	1.34E-03	1.24E-03	1.21E-03	1.09E-04	0.002	0.002	0.1	0.3	7E-06	0.0008	0.0002	0.04	0.0001	0.03
	3-7-0-0	1114.0833	1114.0800	2.92	3.38E-03	3.43E-03	4.98E-03	2.44E-03	3.56E-03	1.06E-03	2.30E-03	2.46E-03	3.21E-03	2.60E-03	2.65E-03	3.96E-04	4.13E-03	3.07E-03	3.15E-03	2.96E-03	3.33E-03	5.39E-04	1.67E-03	1.99E-03	1.64E-03	1.92E-03	1.81E-03	1.78E-04	3.62E-03	2.18E-03	3.28E-03	3.31E-03	3.10E-03	6.28E-04	0.2	0.7	0.02	0.5	0.09	0.008	0.3	0.002	0.6	0.007
	4-3-0-0	828.4468	828.4447	2.53	3.32E-03	3.92E-03	3.89E-03	3.13E-03	3.56E-03	4.02E-04	4.10E-03	2.67E-03	2.79E-03	4.19E-03	3.44E-03	8.17E-04	8.11E-03	7.21E-03	9.45E-03	7.37E-03	8.03E-03	1.02E-03	1.10E-02	9.36E-03	8.41E-03	8.70E-03	9.37E-03	1.18E-03	7.81E-03	9.33E-03	7.57E-03	9.27E-03	8.49E-03	9.31E-04	0.8	0.0002	0.00009	0.00007	0.0004	0.0002	0.0002	0.1	0.5	0.3
	4-3-1-0	915.4914	915.4889	2.73	6.98E-03	8.44E-03	8.29E-03	6.00E-03	7.43E-03	1.16E-03	8.37E-03	5.98E-03	5.73E-03	7.08E-03	6.79E-03	1.21E-03	1.44E-02	1.38E-02	1.88E-02	1.26E-02	1.49E-02	2.71E-03	1.43E-02	1.22E-02	1.08E-02	1.08E-02	1.20E-02	1.63E-03	1.16E-02	1.21E-02	1.10E-02	1.28E-02	1.19E-02	7.78E-04	0.5	0.002	0.004	0.0007	0.002	0.002	0.0004	0.1	0.08	0.9
	4-4-0-0	930.4967	930.4939	3.01	5.74E-03	6.05E-03	6.35E-03	7.50E-03	6.41E-03	7.70E-04	7.30E-03	6.04E-03	6.96E-03	9.79E-03	7.52E-03	1.61E-03	7.22E-03	6.81E-03	7.21E-03	7.09E-03	7.08E-03	1.91E-04	1.53E-02	9.47E-03	1.02E-02	1.06E-02	1.14E-02	2.66E-03	1.06E-02	1.20E-02	9.25E-03	1.01E-02	1.05E-02	1.15E-03	0.3	0.1	0.01	0.001	0.6	0.05	0.02	0.02	0.001	0.5
	4-4-1-0	1111.0836	1111.0808	2.48	2.83E-03	2.74E-03	2.96E-03	1.78E-03	2.58E-03	5.41E-04	7.83E-03	7.53E-03	7.83E-03	7.59E-03	7.70E-03	1.58E-04	4.48E-03	3.87E-03	4.40E-03	3.99E-03	4.18E-03	2.99E-04	2.18E-03	2.31E-03	2.02E-03	2.39E-03	2.23E-03	1.61E-04	3.26E-03	3.19E-03	3.52E-03	3.42E-03	3.35E-03	1.50E-04	2E-06	0.002	0.3	0.03	1E-06	0	0	0.00003	0.002	0.00005
	4-4-1-1	1198.1282	1198.1256	2.13	3.23E-03	3.03E-03	3.26E-03	2.14E-03	2.92E-03	5.30E-04	1.64E-03	1.82E-03	1.75E-03	1.59E-03	1.70E-03	1.06E-04	5.27E-03	5.48E-03	5.38E-03	4.89E-03	5.26E-03	2.57E-04	3.43E-03	3.52E-03	3.30E-03	4.11E-03	3.59E-03	3.60E-04	4.57E-03	4.55E-03	4.36E-03	4.30E-03	4.45E-03	1.37E-04	0.004	0.0002	0.08	0.001	0	0.00006	0	0.0003	0.001	0.004
	4-5-0-0	1032.5466	1032.5437	2.81	3.88E-02	4.00E-02	4.39E-02	4.69E-02	4.24E-02	3.71E-03	3.90E-02	3.68E-02	3.98E-02	4.15E-02	3.93E-02	1.93E-03	3.67E-02	3.21E-02	3.12E-02	3.25E-02	3.31E-02	2.44E-03	4.88E-02	3.27E-02	3.33E-02	3.73E-02	3.80E-02	7.46E-03	3.87E-02	3.34E-02	3.37E-02	3.19E-02	3.44E-02	2.98E-03	0.2	0.006	0.3	0.02	0.007	0.8	0.03	0.3	0.5	0.4
	4-5-0-1	1213.1335	1213.1293	3.42	3.86E-03	4.75E-03	3.95E-03	2.44E-03	3.75E-03	9.62E-04	2.13E-03	2.17E-03	1.92E-03	1.53E-03	1.94E-03	2.95E-04	4.63E-03	4.63E-03	5.11E-03	4.59E-03	4.74E-03	2.45E-04	1.32E-03	2.19E-03	1.94E-03	2.29E-03	1.93E-03	4.35E-04	3.20E-03	2.85E-03	3.40E-03	2.62E-03	3.02E-03	3.50E-04	0.01	0.09	0.01	0.2	6E-06	1	0.003	0.00003	0.0002	0.008
	4-5-0-2	1393.7203	1393.7161	3.01	1.03E-02	8.53E-03	3.70E-03	5.90E-03	7.12E-03	2.92E-03	3.71E-03	5.11E-03	2.36E-03	2.98E-03	3.54E-03	1.18E-03	1.71E-02	2.31E-02	2.48E-02	2.52E-02	2.26E-02	3.78E-03	4.34E-03	7.17E-03	9.60E-03	1.11E-02	8.06E-03	2.97E-03	2.06E-02	2.41E-02	2.74E-02	2.51E-02	2.43E-02	2.84E-03	0.06	0.0006	0.7	0.0001	0.00007	0.03	0.00001	0.0009	0.5	0.0002
	4-5-1-1	1300.1781	1300.1736	3.42	4.90E-03	4.91E-03	4.69E-03	2.72E-03	4.30E-03	1.06E-03	1.98E-03	1.95E-03	1.85E-03	1.75E-03	1.88E-03	1.04E-04	5.00E-03	5.87E-03	6.61E-03	5.12E-03	5.65E-03	7.43E-04	2.01E-03	2.45E-03	2.53E-03	2.64E-03	2.41E-03	2.77E-04	2.99E-03	2.85E-03	3.16E-03	2.59E-03	2.90E-03	2.37E-04	0.004	0.08	0.01	0.04	0.00006	0.01	0.0002	0.0002	0.0004	0.04
	4-5-1-2	1480.7649	1480.7594	3.71	1.51E-02	1.37E-02	5.48E-03	9.95E-03	1.11E-02	4.31E-03	5.70E-03	6.98E-03	2.91E-03	3.95E-03	4.88E-03	1.81E-03	1.12E-02	1.76E-02	1.67E-02	1.88E-02	1.61E-02	3.36E-03	8.31E-03	1.73E-02	1.84E-02	1.79E-02	1.55E-02	4.79E-03	2.21E-02	2.79E-02	2.93E-02	2.76E-02	2.67E-02	3.20E-03	0.04	0.1	0.2	0.001	0.001	0.006	0.00002	0.8	0.004	0.008
	4-6-0-0	1134.5965	1134.5926	3.44	1.04E-02	1.08E-02	1.47E-02	1.56E-02	1.29E-02	2.64E-03	1.06E-02	1.06E-02	1.02E-02	8.06E-03	9.87E-03	1.22E-03	3.91E-03	2.97E-03	4.00E-03	3.69E-03	3.64E-03	4.64E-04	8.84E-03	6.00E-03	6.62E-03	5.74E-03	6.80E-03	1.41E-03	7.64E-03	7.62E-03	7.35E-03	6.60E-03	7.30E-03	4.84E-04	0.09	0.0005	0.007	0.006	0.00008	0.02	0.008	0.005	0.00004	0.5
	4-6-0-1	1315.1834	1315.1792	3.16	1.10E-02	9.10E-03	7.87E-03	8.65E-03	9.16E-03	1.34E-03	4.08E-03	4.48E-03	3.72E-03	3.61E-03	3.97E-03	3.95E-04	4.25E-03	3.91E-03	3.79E-03	4.12E-03	4.02E-03	2.08E-04	2.31E-03	3.91E-03	4.01E-03	4.17E-03	3.60E-03	8.70E-04	5.22E-03	5.44E-03	5.57E-03	4.95E-03	5.30E-03	2.73E-04	0.0003	0.0003	0.0004	0.001	0.9	0.5	0.001	0.4	0.0003	0.01
	4-6-0-2	1495.7702	1495.7633	4.61	2.02E-03	1.86E-03	7.96E-04	1.37E-03	1.51E-03	5.51E-04	5.55E-04	6.35E-04	5.84E-04	7.22E-04	6.24E-04	7.31E-05	6.61E-04	1.65E-03	9.00E-04	1.34E-03	1.14E-03	4.41E-04	3.59E-04	5.47E-04	6.16E-04	7.65E-04	5.71E-04	1.68E-04	8.99E-04	1.00E-03	1.40E-03	1.61E-03	1.23E-03	3.33E-04	0.02	0.3	0.02	0.4	0.06	0.6	0.01	0.05	0.8	0.01
	4-6-1-1	1402.2280	1402.2256	1.68	1.38E-02	1.16E-02	7.77E-03	8.30E-03	1.04E-02	2.85E-03	6.26E-03	6.44E-03	4.60E-03	5.00E-03	5.58E-03	9.10E-04	9.42E-03	1.12E-02	1.05E-02	1.19E-02	1.08E-02	1.05E-03	2.00E-03	6.30E-03	5.31E-03	6.04E-03	4.91E-03	1.99E-03	9.66E-03	8.83E-03	1.21E-02	1.16E-02	1.05E-02	1.55E-03	0.02	0.8	0.02	0.9	0.0003	0.6	0.001	0.002	0.8	0.004
	4-6-1-2	1582.8148	1582.8156	-0.51	2.17E-04	2.36E-04	1.59E-04	2.40E-04	2.13E-04	3.74E-05	2.36E-04	1.99E-04	1.33E-04	1.79E-04	1.87E-04	4.29E-05	2.26E-04	3.02E-04	3.22E-04	3.53E-04	3.01E-04	5.43E-05	2.82E-05	9.67E-05	9.03E-05	1.20E-04	8.39E-05	3.93E-05	2.13E-04	2.78E-04	2.38E-04	1.55E-04	2.21E-04	5.15E-05	0.4	0.04	0.003	0.8	0.02	0.01	0.4	0.0006	0.08	0.005
	4-7-0-1	1417.2333	1417.2308	1.73	7.29E-04	6.06E-04	5.58E-04	1.02E-03	7.29E-04	2.08E-04	8.80E-04	9.11E-04	6.08E-04	5.65E-04	7.41E-04	1.80E-04	4.06E-04	4.51E-04	5.39E-04	5.69E-04	4.91E-04	7.57E-05	1.74E-04	4.24E-04	5.15E-04	4.43E-04	3.89E-04	1.48E-04	5.54E-04	6.92E-04	7.92E-04	6.40E-04	6.70E-04	9.94E-05	0.9	0.08	0.04	0.6	0.04	0.02	0.5	0.3	0.03	0.02
	4-7-1-0	1323.6910	1323.6884	1.96	2.53E-03	2.24E-03	3.42E-03	1.74E-03	2.48E-03	7.05E-04	1.52E-03	1.55E-03	1.58E-03	1.23E-03	1.47E-03	1.61E-04	2.72E-03	2.07E-03	2.89E-03	2.44E-03	2.53E-03	3.60E-04	1.12E-03	1.64E-03	1.54E-03	1.37E-03	1.42E-03	2.29E-04	3.43E-03	2.34E-03	3.53E-03	3.70E-03	3.25E-03	6.17E-04	0.03	0.9	0.03	0.2	0.002	0.7	0.001	0.002	0.09	0.001
	4-7-1-1	1504.2779	1504.2740	2.56	4.59E-04	4.92E-04	3.20E-04	4.15E-04	4.21E-04	7.46E-05	3.08E-04	3.41E-04	2.66E-04	2.69E-04	2.96E-04	3.57E-05	1.03E-04	1.06E-04	1.31E-04	1.09E-04	1.12E-04	1.30E-05	3.29E-05	1.21E-04	1.34E-04	9.58E-05	9.60E-05	4.50E-05	3.11E-04	3.49E-04	4.24E-04	4.19E-04	3.76E-04	5.49E-05	0.02	0.0002	0.0003	0.4	0.00007	0.0004	0.05	0.5	0.00009	0.0002
	5-3-0-0	951.0100	951.0072	2.89	2.82E-02	2.88E-02	2.78E-02	3.61E-02	3.02E-02	3.91E-03	6.50E-02	5.38E-02	6.28E-02	8.75E-02	6.73E-02	1.43E-02	5.07E-02	5.25E-02	4.54E-02	5.49E-02	5.09E-02	4.03E-03	6.08E-02	4.97E-02	4.86E-02	5.45E-02	5.34E-02	5.58E-03	5.51E-02	5.10E-02	4.61E-02	4.80E-02	5.00E-02	3.90E-03	0.002	0.0003	0.0005	0.0004	0.07	0.1	0.06	0.5	0.8	0.4
	5-3-1-0	1038.0546	1038.0514	3.03	2.60E-02	2.83E-02	2.53E-02	3.17E-02	2.78E-02	2.87E-03	4.01E-02	3.64E-02	4.35E-02	5.82E-02	4.46E-02	9.58E-03	4.20E-02	4.33E-02	3.83E-02	4.61E-02	4.24E-02	3.25E-03	5.53E-02	4.21E-02	4.33E-02	4.61E-02	4.67E-02	5.99E-03	4.52E-02	3.97E-02	3.53E-02	3.57E-02	3.90E-02	4.59E-03	0.02	0.0005	0.001	0.006	0.7	0.7	0.3	0.3	0.3	0.09
	5-4-0-0	1053.0599	1053.0565	3.18	2.54E-02	2.86E-02	2.83E-02	2.76E-02	2.75E-02	1.47E-03	3.35E-02	3.02E-02	3.08E-02	3.63E-02	3.27E-02	2.80E-03	2.79E-02	2.45E-02	2.92E-02	2.64E-02	2.70E-02	2.03E-03	4.16E-02	3.10E-02	3.15E-02	3.44E-02	3.46E-02	4.88E-03	2.69E-02	2.72E-02	2.54E-02	2.63E-02	2.64E-02	7.81E-04	0.02	0.7	0.03	0.3	0.02	0.5	0.005	0.03	0.6	0.02
	5-4-0-1	1233.6467	1233.6422	3.65	4.57E-02	4.41E-02	4.42E-02	4.26E-02	4.41E-02	1.24E-03	8.50E-02	7.89E-02	7.37E-02	7.34E-02	7.78E-02	5.41E-03	9.94E-02	9.70E-02	9.53E-02	9.57E-02	9.68E-02	1.87E-03	1.02E-01	8.53E-02	8.70E-02	8.81E-02	9.06E-02	7.63E-03	1.12E-01	1.03E-01	1.01E-01	9.83E-02	1.04E-01	6.10E-03	0.00002	0	0.00002	1E-06	0.0006	0.03	0.0007	0.2	0.07	0.03
	5-4-1-0	1140.1045	1140.1008	3.20	2.77E-02	3.04E-02	2.93E-02	2.94E-02	2.92E-02	1.11E-03	3.78E-02	3.42E-02	3.62E-02	4.27E-02	3.77E-02	3.64E-03	2.78E-02	2.68E-02	2.63E-02	2.66E-02	2.69E-02	6.15E-04	3.98E-02	2.97E-02	3.22E-02	3.08E-02	3.31E-02	4.54E-03	1.96E-02	2.10E-02	1.84E-02	1.87E-02	1.94E-02	1.16E-03	0.004	0.01	0.1	0.00002	0.001	0.2	0.00007	0.03	0.00003	0.001

5-4-1-1	1320.6913	1320.6877	2.73	4.40E-02	4.45E-02	4.63E-02	4.13E-02	4.40E-02	2.06E-03	3.88E-02	3.75E-02	3.98E-02	4.21E-02	3.96E-02	1.94E-03	7.58E-02	7.57E-02	7.33E-02	7.19E-02	7.42E-02	1.91E-03	8.94E-02	7.39E-02	7.78E-02	7.62E-02	7.93E-02	6.94E-03	9.43E-02	8.85E-02	8.23E-02	7.32E-02	8.46E-02	9.01E-03	0.02	1E-06	0.00007	0.0001	0	0.00003	0.00007	0.2	0.06	0.4
5-5-0-1	1335.6966	1335.6928	2.84	2.74E-02	2.63E-02	2.77E-02	2.53E-02	2.67E-02	1.09E-03	1.87E-02	2.04E-02	1.81E-02	1.49E-02	1.80E-02	2.30E-03	2.13E-02	2.50E-02	1.97E-02	2.05E-02	2.16E-02	2.37E-03	1.83E-02	1.86E-02	1.82E-02	2.02E-02	1.88E-02	9.30E-04	2.36E-02	2.16E-02	2.33E-02	1.88E-02	2.18E-02	2.20E-03	0.0005	0.008	0.00003	0.008	0.07	0.5	0.05	0.07	0.9	0.05
5-5-0-2	1516.2835	1516.2784	3.33	1.25E-02	9.22E-03	3.25E-03	8.66E-03	8.42E-03	3.85E-03	9.16E-03	1.14E-02	3.50E-03	5.70E-03	7.43E-03	3.50E-03	7.14E-03	1.30E-02	7.78E-03	1.33E-02	1.03E-02	3.31E-03	1.66E-03	8.16E-03	8.21E-03	1.02E-02	7.05E-03	3.71E-03	9.74E-03	1.23E-02	1.41E-02	1.47E-02	1.27E-02	2.24E-03	0.7	0.5	0.6	0.1	0.3	0.9	0.04	0.2	0.3	0.04
5-5-1-0	1242.1544	1242.1511	2.62	6.05E-02	6.60E-02	7.35E-02	6.12E-02	6.53E-02	6.01E-03	6.39E-02	5.68E-02	5.82E-02	5.98E-02	5.97E-02	3.10E-03	5.08E-02	4.58E-02	4.95E-02	4.42E-02	4.76E-02	3.08E-03	4.19E-02	3.93E-02	3.70E-02	3.77E-02	3.90E-02	2.18E-03	4.31E-02	4.27E-02	4.17E-02	4.21E-02	4.24E-02	5.95E-04	0.1	0.002	0.0002	0.0003	0.001	0.00003	0.00003	0.004	0.02	0.02
5-5-1-2	1603.3281	1603.3232	3.02	4.53E-02	3.60E-02	1.03E-02	2.96E-02	3.03E-02	1.48E-02	2.24E-02	2.57E-02	9.27E-03	1.59E-02	1.83E-02	7.28E-03	2.26E-02	4.23E-02	2.85E-02	4.16E-02	3.38E-02	9.74E-03	5.04E-03	3.58E-02	3.70E-02	3.84E-02	2.91E-02	1.61E-02	3.27E-02	4.09E-02	4.77E-02	4.90E-02	4.26E-02	7.47E-03	0.2	0.7	0.9	0.2	0.04	0.3	0.004	0.6	0.2	0.2
5-6-0-0	1257.1597	1257.1548	3.86	9.57E-03	9.93E-03	1.29E-02	1.14E-02	1.10E-02	1.52E-03	9.05E-03	1.31E-02	1.01E-02	7.46E-03	9.93E-03	2.40E-03	4.75E-03	6.28E-03	3.81E-03	4.11E-03	4.74E-03	1.10E-03	4.01E-03	2.94E-03	2.81E-03	2.97E-03	3.18E-03	5.56E-04	4.86E-03	3.74E-03	4.23E-03	4.34E-03	4.29E-03	4.59E-04	0.5	0.0006	0.00007	0.0002	0.008	0.002	0.004	0.05	0.5	0.02
5-6-0-3	1199.6161	1199.6120	3.42	1.31E-03	1.11E-03	1.86E-04	8.98E-04	8.76E-04	4.91E-04	3.48E-04	4.30E-04	7.68E-04	4.94E-04	5.10E-04	1.82E-04	1.98E-04	2.72E-04	3.29E-04	2.45E-04	2.61E-04	5.47E-05	1.32E-04	5.23E-04	4.83E-04	7.83E-04	4.80E-04	2.67E-04	2.40E-04	4.49E-04	5.35E-04	1.03E-03	5.64E-04	3.36E-04	0.2	0.05	0.2	0.3	0.04	0.9	0.8	0.2	0.1	0.7
5-6-1-0	896.4721	896.4689	3.61	1.31E-02	1.44E-02	1.69E-02	1.30E-02	1.43E-02	1.83E-03	1.35E-02	1.29E-02	1.38E-02	1.16E-02	1.29E-02	9.89E-04	8.48E-03	6.79E-03	8.55E-03	6.44E-03	7.56E-03	1.11E-03	5.57E-03	6.59E-03	5.80E-03	5.94E-03	5.98E-03	4.37E-04	7.89E-03	7.54E-03	8.17E-03	7.98E-03	7.89E-03	2.66E-04	0.2	0.0007	0.0001	0.0004	0.0003	0.00001	0.00006	0.04	0.6	0.0003
5-6-1-3	1257.6458	1257.6420	3.05	4.16E-04	4.15E-04	1.25E-04	5.15E-04	3.68E-04	1.68E-04	6.44E-04	6.12E-04	5.02E-04	5.81E-04	5.84E-04	6.09E-05	1.27E-04	3.40E-04	1.63E-04	3.68E-04	2.50E-04	1.22E-04	8.38E-05	4.16E-04	5.54E-04	4.94E-04	3.87E-04	2.10E-04	2.92E-04	3.54E-04	6.13E-04	1.43E-03	6.72E-04	5.24E-04	0.05	0.3	0.9	0.3	0.003	0.1	0.8	0.3	0.2	0.4
5-6-2-0	954.5019	954.5024	-0.56	3.45E-03	3.08E-03	3.87E-03	3.64E-03	3.51E-03	3.34E-04	2.75E-03	3.32E-03	3.00E-03	2.41E-03	2.87E-03	3.86E-04	3.56E-03	3.14E-03	1.82E-03	2.96E-03	2.87E-03	7.43E-04	1.48E-03	1.16E-03	1.25E-03	1.88E-03	1.44E-03	3.22E-04	2.65E-03	1.31E-03	2.39E-03	1.11E-03	1.87E-03	7.69E-04	0.05	0.2	0.0001	0.008	1	0.001	0.06	0.01	0.1	0.4
5-6-2-1	1074.8931	1074.8932	-0.09	9.68E-03	7.50E-03	1.84E-03	6.61E-03	6.41E-03	3.31E-03	5.18E-03	6.48E-03	2.29E-03	3.65E-03	4.40E-03	1.82E-03	5.00E-03	9.14E-03	4.03E-03	8.18E-04	4.75E-03	3.43E-03	8.22E-04	4.08E-03	4.06E-03	5.71E-03	3.67E-03	2.05E-03	5.35E-03	4.61E-03	6.62E-03	6.45E-03	5.76E-03	9.51E-04	0.3	0.5	0.2	0.7	0.9	0.6	0.2	0.6	0.6	0.1
5-7-0-0	906.4757	906.4728	3.16	5.34E-03	4.86E-03	6.55E-03	4.68E-03	5.36E-03	8.43E-04	3.45E-03	3.39E-03	3.31E-03	2.79E-03	3.24E-03	3.01E-04	2.92E-03	2.28E-03	3.00E-03	2.35E-03	2.64E-03	3.75E-04	1.69E-03	2.14E-03	2.09E-03	1.89E-03	1.95E-03	2.05E-04	4.16E-03	3.44E-03	4.18E-03	4.39E-03	4.04E-03	4.16E-04	0.003	0.001	0.0002	0.03	0.05	0.0004	0.02	0.02	0.002	0.0001
5-7-0-1	1026.8669	1026.8636	3.21	7.16E-03	5.72E-03	4.66E-03	7.16E-03	6.18E-03	1.22E-03	5.30E-03	1.05E-02	5.02E-03	3.84E-03	6.16E-03	2.95E-03	2.53E-03	5.46E-03	1.90E-03	3.34E-03	3.31E-03	1.55E-03	8.88E-04	2.88E-03	2.62E-03	2.91E-03	2.32E-03	9.66E-04	3.04E-03	2.82E-03	3.22E-03	3.41E-03	3.12E-03	2.54E-04	1	0.03	0.003	0.003	0.1	0.05	0.09	0.3	0.8	0.2
5-7-1-0	964.5054	964.5031	2.38	2.01E-02	1.85E-02	2.31E-02	1.73E-02	1.98E-02	2.53E-03	1.40E-02	1.35E-02	1.41E-02	1.29E-02	1.36E-02	5.53E-04	1.32E-02	1.12E-02	1.37E-02	1.06E-02	1.22E-02	1.53E-03	7.21E-03	1.01E-02	9.96E-03	8.27E-03	8.89E-03	1.40E-03	1.87E-02	1.69E-02	1.91E-02	2.01E-02	1.87E-02	1.36E-03	0.003	0.002	0.0003	0.5	0.1	0.0008	0.0004	0.02	0.0007	0.00006
5-8-1-1	1152.9299	1152.9266	2.86	6.29E-03	4.64E-03	8.85E-04	3.00E-03	3.71E-03	2.31E-03	2.58E-03	3.24E-03	1.01E-03	1.81E-03	2.16E-03	9.63E-04	1.55E-03	3.25E-03	1.67E-03	3.41E-03	2.47E-03	9.95E-04	1.95E-04	3.92E-03	3.03E-03	4.83E-03	2.99E-03	2.01E-03	1.09E-03	1.47E-03	2.34E-03	3.85E-03	2.19E-03	1.23E-03	0.3	0.4	0.7	0.3	0.7	0.5	1	0.7	0.7	0.5
6-3-0-0	1073.5731	1073.5691	3.73	8.30E-04	9.48E-04	7.52E-04	1.21E-03	9.35E-04	2.00E-04	1.37E-03	1.26E-03	2.16E-03	3.13E-03	1.98E-03	8.64E-04	1.84E-03	2.51E-03	1.88E-03	2.83E-03	2.27E-03	4.87E-04	4.93E-03	3.32E-03	3.41E-03	3.14E-03	3.70E-03	8.28E-04	1.25E-03	1.14E-03	1.08E-03	1.44E-03	1.23E-03	1.59E-04	0.06	0.002	0.0006	0.06	0.6	0.03	0.1	0.02	0.007	0.001
6-3-1-0	1160.6177	1160.6133	3.79	1.57E-03	1.60E-03	1.74E-03	4.33E-04	1.33E-03	6.06E-04	2.07E-03	2.92E-03	2.68E-03	3.50E-03	2.79E-03	5.92E-04	1.85E-03	2.42E-03	2.22E-03	2.79E-03	2.32E-03	3.90E-04	5.49E-03	3.88E-03	3.85E-03	3.75E-03	4.24E-03	8.32E-04	2.48E-03	2.19E-03	1.78E-03	1.92E-03	2.09E-03	3.05E-04	0.01	0.03	0.001	0.07	0.2	0.03	0.08	0.006	0.4	0.003
6-4-0-0	1175.6230	1175.6190	3.40	7.59E-04	8.27E-04	8.84E-04	8.45E-04	8.29E-04	5.24E-05	1.49E-03	1.64E-03	2.11E-03	2.62E-03	1.96E-03	5.09E-04	1.42E-03	1.38E-03	1.71E-03	1.44E-03	1.49E-03	1.49E-04	5.41E-03	3.76E-03	4.23E-03	3.76E-03	4.29E-03	7.77E-04	2.03E-03	2.16E-03	2.05E-03	1.96E-03	2.05E-03	8.37E-05	0.004	0.0002	0.0001	0	0.1	0.002	0.7	0.0004	0.0006	0.001
6-4-0-1	1356.2099	1356.2050	3.58	4.91E-03	4.80E-03	5.24E-03	4.29E-03	4.81E-03	3.93E-04	3.59E-03	4.51E-03	3.79E-03	4.08E-03	3.99E-03	4.02E-04	5.37E-03	5.73E-03	5.35E-03	5.87E-03	5.58E-03	2.60E-04	2.08E-02	1.32E-02	1.50E-02	1.30E-02	1.55E-02	3.65E-03	7.08E-03	8.44E-03	5.83E-03	6.27E-03	6.91E-03	1.15E-03	0.03	0.02	0.001	0.01	0.0006	0.0008	0.003	0.002	0.07	0.004
6-4-1-0	1262.6676	1262.6631	3.56	2.73E-04	2.27E-04	2.52E-04	2.81E-04	2.58E-04	2.41E-05	1.04E-03	1.00E-03	1.28E-03	2.17E-03	1.37E-03	5.44E-04	1.75E-03	1.85E-03	1.37E-03	2.09E-03	1.76E-03	2.98E-04	5.60E-03	3.26E-03	3.98E-03	3.35E-03	4.05E-03	1.09E-03	1.80E-03	1.77E-03	1.58E-03	1.57E-03	1.68E-03	1.21E-04	0.006	0.00006	0.0004	0	0.3	0.005	0.3	0.007	0.6	0.005
6-4-1-1	1443.2545	1443.2493	3.57	6.71E-03	7.32E-03	7.64E-03	6.22E-03	6.97E-03	6.32E-04	6.59E-03	6.84E-03	6.96E-03	8.16E-03	7.13E-03	6.98E-04	7.38E-03	8.33E-03	8.61E-03	8.38E-03	8.17E-03	5.44E-04	1.61E-02	1.27E-02	1.34E-02	1.14E-02	1.34E-02	1.95E-03	9.85E-03	9.35E-03	8.07E-03	7.86E-03	8.78E-03	9.71E-04	0.7	0.03	0.0008	0.02	0.06	0.0009	0.03	0.002	0.3	0.005
6-5-0-0	1277.6729	1277.6710	1.49	5.38E-03	6.06E-03	6.72E-03	6.00E-03	6.04E-03	5.49E-04	7.95E-03	7.35E-03	1.14E-02	1.82E-03	7.13E-03	3.97E-03	7.56E-03	7.70E-03	6.61E-03	6.79E-03	7.16E-03	5.44E-04	1.02E-02	7.44E-03	7.14E-03	7.47E-03	8.06E-03	1.43E-03	2.44E-03	2.83E-03	1.92E-03	2.47E-03	2.42E-03	3.75E-04	0.6	0.03	0.04	0.00004	1	0.7	0.06	0.3	7E-06	0.0003
6-5-0-1	1458.2598	1458.2554	2.98	2.90E-03	2.82E-03	3.36E-03	2.82E-03	2.98E-03	2.61E-04	2.94E-03	2.87E-03	3.41E-03	2.95E-03	3.04E-03	2.49E-04	3.22E-03	3.67E-03	3.47E-03	3.75E-03	3.53E-03	2.37E-04	6.86E-03	5.99E-03	6.33E-03	5.75E-03	6.23E-03	4.84E-04	2.29E-03	2.24E-03	2.11E-03	1.94E-03	2.14E-03	1.57E-04	0.7	0.02	0.00002	0.002	0.03	0.00002	0.0009	0.00006	0.00007	4E-06
6-5-0-2	1638.8466	1638.8401	3.97	7.30E-04	5.78E-04	4.85E-04	7.86E-04	6.44E-04	1.38E-04	1.25E-03	1.30E-03	7.90E-04	1.15E-03	1.12E-03	2.30E-04	5.68E-03	7.46E-03	6.49E-03	7.59E-03	6.80E-03	8.95E-04	7.83E-03	1.59E-02	1.66E-02	1.51E-02	1.39E-02	4.07E-03	8.52E-03	9.03E-03	9.22E-03	9.52E-03	9.07E-03	4.23E-04	0.01	0.00001	0.0006	0	0.00002	0.0008	0	0.01	0.004	0.06
6-5-1-0	1364.7175	1364.7133	3.08	3.16E-03	3.39E-03	4.54E-03	3.89E-03	3.75E-03	6.09E-04	5.08E-03	4.56E-03	8.25E-03	8.07E-03	6.49E-03	1.94E-03	4.01E-03	4.11E-03	4.47E-03	3.47E-03	4.01E-03	4.10E-04	1.21E-02	9.83E-03	9.18E-03	8.11E-03	9.79E-03	1.67E-03	5.66E-03	6.68E-03	4.59E-03	5.67E-03	5.65E-03	8.52E-04	0.04	0.5	0.0005	0.01	0.05	0.04	0.5	0.0005	0.01	0.004
6-5-1-1	1545.3044	1545.2999	2.88	1.51E-03	1.70E-03	1.95E-03	1.70E-03	1.72E-03	1.81E-04	2.67E-03	2.41E-03	2.60E-03	3.37E-03	2.76E-03	4.21E-04	3.67E-03	4.19E-03	4.46E-03	4.37E-03	4.17E-03	3.54E-04	9.65E-03	1.11E-02	1.18E-02	9.15E-03	1.04E-02	1.23E-03	6.15E-03	5.91E-03	5.66E-03	4.93E-03	5.66E-03	5.29E-04	0.004	0.00002	0.000008	8E-06	0.002	0.00002	0.0001	0.00007	0.003	0.0004
6-5-1-2	1725.8912	1725.8843	4.00	6.49E-03	5.83E-03	3.54E-03	5.17E-03	5.26E-03	1.27E-03	5.09E-03	5.87E-03	2.92E-03	4.07E-03	4.49E-03	1.28E-03	6.78E-03	1.08E-02	8.27E-03	1.12E-02	9.27E-03	2.11E-03	3.89E-03	1.29E-02	1.45E-02	1.32E-02	1.11E-02	4.87E-03	1.03E-02	1.12E-02	1.19E-02	1.23E-02	1.14E-02	8.58E-04	0.4	0.02	0.06	0.0002	0.008	0.04	0.0001	0.5	0.1	0.9
6-6-0-0	920.1512	920.1486	2.79	5.17E-03	5.69E-03	6.80E-03	9.20E-03	6.71E-03	1.79E-03	5.30E-03	5.02E-03	7.55E-03	5.90E-03	5.94E-03	1.13E-03	6.22E-03	4.93E-03	5.82E-03	4.39E-03	5.34E-03	8.31E-04	4.52E-03	4.18E-03	3.88E-03	3.84E-03	4.11E-03	3.17E-04	2.46E-03	2.82E-03	2.59E-03	2.63E-03	2.63E-03	1.48E-04	0.5	0.2	0.03	0.004	0.4	0.02	0.001	0.03	0.0007	0.0001
6-6-0-1	1040.5424	1040.5393	2.98	6.54E-03	6.26E-03	7.12E-03	6.59E-03	6.63E-03	3.57E-04	7.49E-03	7.17E-03	1.14E-02	9.63E-03	8.91E-03	1.97E-03	1.61E-03	1.82E-03	1.50E-03	1.56E-03	1.62E-03	1.40E-04	3.76E-03	5.37E-03	5.17E-03	4.95E-03	4.81E-03	7.23E-04	3.84E-03	4.45E-03	3.60E-03	4.34E-03	4.06E-03	4.03E-04	0.06	0	0.004	0.00008	0.0003	0.008	0.003	0.0001	0.00003	0.1
6-6-0-2	1160.9336	1160.9299	3.22	2.89E-03	2.51E-03	1.12E-03	2.40E-03	2.23E-03	7.70E-04	1.10E-03	1.40E-03	6.30E-04	9.71E-04	1.02E-03	3.18E-04	1.25E-03	3.01E-03	1.63E-03	2.73E-03	2.15E-03	8.44E-04	6.52E-04	4.05E-03	3.63E-03	4.17E-03	3.12E-03	1.66E-03	2.06E-03	2.44E-03	3.05E-03	3.72E-03	2.82E-03	7.28E-04	0.03	0.9	0.4	0.3	0.05	0.05	0.004	0.3	0.3	0.7
6-6-1-0	978.1809	978.1783	2.66	4.41E-03	4.65E-03	7.91E-03	5.19E-03	5.54E-03	1.61E-03	7.01E-03	6.69E-03	1.00E-02	9.04E-03	8.19E-03	1.60E-03	5.07E-03	3.57E-03	4.78E-03	3.42E-03	4.21E-03	8.37E-04	4.52E-03	4.11E-03	3.31E-03	3.19E-03	3.78E-03	6.41E-04	4.21E-03	3.64E-03	4.05E-03	3.56E-03	3.87E-03	3.15E-04	0.06	0.2	0.09	0.09	0.005	0.002	0.002	0.4	0.5	0.8
6-6-1-1	1098.5721	1098.5681	3.67	1.14E-02	1.00E-02	9.43E-03	1.02E-02	1.03E-02	8.39E-04	9.73E-03	9.86E-03	9.27E-03	1.06E-02	9.87E-03	5.54E-04	9.17E-03	1.02E-02	9.88E-03	9.92E-03	9.81E-03	4.52E-04	6.34E-03	1.19E-02	1.23E-02	1.10E-02	1.04E-02	2.76E-03	9.88E-03	1.22E-02	9.74E-03	1.09E-02	1.07E-02	1.12E-03	0.5	0.4	0.9	0.6	0.9	0.7	0.3	0.7	0.2	0.9
6-6-1-2	1218.9634	1218.9595	3.17	1.88E-03	1.93E-03	3.74E-04	1.57E-03	1.44E-03	7.26E-04	9.89E-04	9.74E-04	4.62E-04	8.51E-04	8.19E-04	2.46E-04	5.81E-04	1.85E-03	1.01E-03	2.06E-03	1.38E-03	6.96E-04	5.29E-04	5.57E-03	4.97E-03	5.19E-03	4.07E-03	2.37E-03	1.21E-03	1.63E-03	2.25E-03	3.06E-03	2.04E-03	8.04E-04	0.2	0.9	0.08	0.3	0.2	0.03	0.03	0.07	0.3	0.2
6-6-1-3	1339.3546	1339.3510	2.69	5.88E-04	6.41E-04	1.70E-04	4.28E-04	4.57E-04	2.11E-04	1.06E-04	1.41E-04	3.65E-05	6.48E-05	8.72E-05	4.60E-05	1.69E-04	1.07E-03	2.61E-04	1.09E-03	6.48E-04	5.01E-04	7.95E-05	1.49E-03	1.25E-03	2.71E-03	1.38E-03	1.08E-03	8.63E-04	9.07E-03	1.22E-03	3.50E-03	3.66E-03	3.79E-03	0.01	0.5	0.1	0.1	0.07	0.05	0.1	0.3	0.2	0.3
6-7-0-0	988.1844	988.1821	2.36	6.88E-03	6.75E-03	9.42E-03	6.14E-03	7.30E-03	1.45E-03	5.61E-03	6.09E-03	8.04E-03	6.01E-03	6.44E-03	1.09E-03	8.17E-03	6.43E-03	6.04E-03	5.69E-03	6.58E-03	1.10E-03	5.53E-03	4.76E-03	4.86E-03	4.61E-03	4.94E-03	4.09E-04	2.31E-03	2.33E-03	2.00E-03	2.30E-03	2.24E-03	1.55E-04	0.4	0.5	0.02	0.0004	0.9	0.04	0.0003	0.03	0.0002	0.00002
6-7-1-0	1046.2142	1046.2117	2.36	1.24E-02	1.08E-02	1.62E-02	1.05E-02	1.25E-02	2.64E-03	1.03E-02	1.02E-02	1.57E-02	1.26E-02	1.22E-02	2.57E-03	1.37E-02	9.72E-03	1.17E-02	9.04E-03	1.10E-02	2.10E-03	8.44E-03	9.89E-03	9.71E-03	1.77E-03	7.45E-03	3.84E-03	5.66E-03	6.40E-03	5.60E-03	5.95E-03	5.90E-03	3.64E-04	0.9	0.4	0.07	0.003	0.5	0.09	0.003	0.2	0.003	0.5
6-7-1-2	1286.9966	1286.9921	3.52	6.99E-03	6.24E-03	1.21E-03	4.17E-03	4.65E-03	2.59E-03	3.18E-03	3.71E-03	1.11E-03	1.57E-03	2.39E-03	1.25E-03	6.40E-04	2.08E-03	1.23E-03	2.06E-03	1.50E-03	7.01E-04	4.11E-04	4.36E-03	3.39E-03	4.65E-03	3.20E-03	1.94E-03	1.20E-03	1.86E-03	2.57E-03	5.26E-03	2.72E-03	1.78E-03	0.2	0.06	0.4	0.3	0.3	0.5	0.8	0.2	0.3	0.7
6-7-1-4	1527.7791	1527.7741	3.27	6.45E-04	5.65E-04	1.26E-05	1.16E-04	3.35E-04	3.17E-04	2.70E-04	2.90E-04	5.21E-05	1.42E-04	1.88E-04	1.12E-04	0.00E+00	0.00E+00	#######	#######	0.00E+00	0.00E+00	0.00E+00	0.00E+00	0.00E+00	0.00E+00	0.00E+00	0.00E+00	0.00E+00	0.00E+00	0.00E+00	0.00E+00	0.00E+00	0.00E+00	0.4	0.08	0.08	0.3	0.02	0.02	0.6	#DIV/0!	0.4	0.4
6-8-0-0	1056.2177	1056.2148	2.75	1.01E-03	1.09E-03	1.09E-03	1.09E-02	3.52E-03	4.92E-03	5.12E-04	1.18E-03	1.06E-03	6.60E-04	8.54E-04	3.20E-04	7.95E-04	7.04E-04	6.89E-04	5.89E-04	6.94E-04	8.43E-05	6.05E-04	6.82E-04	6.91E-04	6.48E-04	6.57E-04	3.89E-05	3.75E-04	3.93E-04	3.53E-04	4.24E-04	3.86E-04	3.01E-05	0.3	0.3	0.3	0.2	0.4	0.3	0.03	0.4	0.0005	0.00003
6-8-0-1	1176.6089	1176.6054	3.00	2.56E-03	1.70E-03	5.46E-04	1.61E-03	1.60E-03	8.25E-04	9.22E-04	1.03E-03	4.91E-04	7.91E-04	8.08E-04	2.32E-04	9.50E-04	1.58E-03	1.10E-03	1.67E-03	1.32E-03	3.52E-04	4.41E-04	2.05E-03	2.05E-03	2.01E-03	1.64E-03	7.99E-04	7.09E-04	9.95E-04	1.15E-03	1.47E-03	1.08E-03	3.19E-04	0.1	0.6	1	0.3	0.05	0.09	0.2	0.5	0.3	0.2
6-8-1-0	1114.2474	1114.2442	2.90	5.28E-03	4.66E-03	4.84E-03	4.87E-03	4.91E-03	2.60E-04	3.39E-03	3.77E-03	4.12E-03	3.49E-03	3.69E-03	3.25E-04	3.12E-03	2.11E-03	2.26E-03	1.85E-03	2.34E-03	5.52E-04	1.29E-03	2.35E-03	2.33E-03	2.13E-03	2.03E-03	4.98E-04	1.50E-03	1.61E-03	1.81E-03	1.88E-03	1.70E-03	1.77E-04	0.001	0.0002	0.00005	1E-06	0.005	0.001	0.00004	0.4	0.07	0.3
6-8-1-1	1234.6387	1234.6350	2.97	1.37E-02	9.77E-03	2.09E-03	7.20E-03	8.18E-03	4.86E-03	5.28E-03	6.62E-03	1.85E-03	3.98E-03	4.43E-03	2.03E-03	2.89E-03	5.62E-03	2.81E-03	5.28E-03	4.15E-03	1.51E-03	4.38E-04	5.96E-03	5.47E-03	7.21E-03	4.77E-03	2.98E-03	2.45E-03	3.24E-03	1.13E-03	5.92E-03	3.18E-03	2.02E-03	0.2	0.2	0.3	0.1	0.8	0.9	0.4	0.7	0.5	0.4
6-9-0-0	1124.2510	1124.2474	3.17	7.54E-04	6.12E-04	6.53E-04	6.91E-04	6.77E-04	6.02E-05	5.10E-04	5.50E-04	4.93E-04	5.45E-04	5.25E-04	2.72E-05	5.35E-04	4.24E-04	4.94E-04	4.21E-04	4.69E-04	5.60E-05	2.46E-04	4.50E-04	4.15E-04	3.60E-04	3.68E-04	8.92E-05	4.32E-04	4.92E-04	4.70E-04	5.88E-04	4.96E-04	6.67E-05	0.004	0.002	0.001	0.007	0.1	0.02	0.5	0.1	0.6	0.06
6-9-1-0	1182.2807	1182.2768	3.30	3.32E-03	2.44E-03	2.19E-03	2.24E-03	2.55E-03	5.27E-04	1.83E-03	1.99E-03	1.96E-03	2.39E-03	2.04E-03	2.39E-04	2.73E-03	2.14E-03	2.16E-03	2.14E-03	2.29E-03	2.90E-04	8.93E-04	2.69E-03	2.69E-03	2.21E-03	2.12E-03	8.50E-04	1.68E-03	1.75E-03	1.98E-03	2.29E-03	1.92E-03	2.73E-04	0.1	0.4	0.4	0.08	0.2	0.9	0.5	0.7	0.1	0.7
7-4-1-1	1044.2144	1044.2105	3.70	6.86E-04	6.94E-04	5.80E-04	6.38E-04	6.50E-04	5.25E-05	6.77E-04	7.17E-04	9.45E-04	1.21E-03	8.87E-04	2.45E-04	9.00E-04	1.30E-03	1.32E-03	1.33E-03	1.21E-03	2.07E-04	2.16E-03	1.72E-03	1.98E-03	1.55E-03	1.85E-03	2.73E-04	8.28E-04	1.07E-03	8.82E-04	8.76E-04	9.13E-04	1.05E-04	0.1	0.002	0.0001	0.004	0.09	0.002	0.9	0.01	0.04	0.0007
7-5-0-0	933.8267	933.8239	2.96	1.85E-04	2.04E-04	1.47E-04	2.19E-04	1.88E-04	3.10E-05	5.54E-04	3.89E-04	8.67E-04	1.23E-03	7.59E-04	3.69E-04	2.83E-04	3.28E-04	3.40E-04	3.33E-04	3.21E-04	2.58E-05	1.21E-03	1.46E-03	1.37E-03	1.64E-03	1.42E-03	1.76E-04	1.32E-03	8.87E-04	1.46E-03	1.25E-03	1.23E-03	2.45E-04	0.02	0.0006	0.00001	0.0002	0.06	0.02	0.08	0.00002	0.0003	0.3

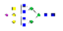	7-5-0-1	1054.2179	1054.2145	3.23	9.12E-04	9.07E-04	1.10E-03	8.65E-04	9.47E-04	1.07E-04	2.05E-03	1.82E-03	2.77E-03	3.71E-03	2.59E-03	8.50E-04	1.27E-03	1.32E-03	1.54E-03	1.49E-03	1.41E-03	1.32E-04	3.81E-03	2.36E-03	3.56E-03	2.74E-03	3.12E-03	6.79E-04	7.13E-04	8.39E-04	8.07E-04	7.40E-04	7.75E-04	5.83E-05	0.009	0.002	0.0007	0.03	0.03	0.4	0.005	0.003	0.0001	0.0005
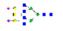	7-5-0-2	1174.6091	1174.6055	3.09	8.10E-04	7.99E-04	6.29E-04	6.94E-04	7.33E-04	8.67E-05	1.66E-04	1.62E-04	8.23E-05	1.32E-04	1.36E-04	3.86E-05	4.38E-04	7.60E-04	6.86E-04	7.67E-04	6.63E-04	1.54E-04	7.35E-04	6.56E-04	7.55E-04	5.59E-04	6.76E-04	8.91E-05	2.67E-04	3.34E-04	3.42E-04	3.42E-04	3.22E-04	3.66E-05	0.00002	0.5	0.4	0.0001	0.0006	0.00003	0.0004	0.9	0.005	0.0003
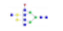	7-5-1-0	991.8564	991.8533	3.13	2.58E-04	2.93E-04	3.05E-04	3.34E-04	2.98E-04	3.17E-05	8.66E-04	8.61E-04	1.63E-03	2.51E-03	1.47E-03	7.85E-04	9.38E-04	1.31E-03	1.24E-03	1.41E-03	1.22E-03	2.02E-04	4.29E-03	3.97E-03	4.19E-03	3.14E-03	3.90E-03	5.23E-04	1.32E-03	1.99E-03	2.00E-03	1.91E-03	1.81E-03	3.26E-04	0.02	0.0001	0.00001	0.00009	0.6	0.002	0.5	0.00008	0.02	0.0005
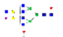	7-5-1-1	1112.2476	1112.2441	3.18	5.17E-04	5.23E-04	6.84E-04	7.92E-04	6.29E-04	1.34E-04	5.09E-04	5.33E-04	4.84E-04	7.74E-04	5.75E-04	1.34E-04	8.49E-04	9.26E-04	9.08E-04	1.11E-03	9.48E-04	1.14E-04	3.07E-03	3.29E-03	4.01E-03	2.92E-03	3.32E-03	4.83E-04	1.22E-04	2.18E-04	1.21E-04	1.41E-04	1.51E-04	4.61E-05	0.6	0.01	0.00004	0.0005	0.005	0.00003	0.001	0.00007	0.00001	0.00001
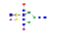	7-5-1-2	1232.6389	1232.6366	1.84	8.53E-04	7.74E-04	3.04E-04	6.35E-04	6.42E-04	2.42E-04	5.27E-04	5.86E-04	2.26E-04	4.55E-04	4.48E-04	1.58E-04	1.30E-03	2.36E-03	1.95E-03	2.60E-03	2.05E-03	5.71E-04	1.02E-03	3.82E-03	4.05E-03	3.20E-03	3.03E-03	1.38E-03	2.95E-04	4.51E-04	3.56E-04	4.60E-04	3.90E-04	7.93E-05	0.2	0.004	0.01	0.1	0.002	0.01	0.5	0.2	0.001	0.009
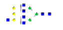	7-6-0-0	1001.8599	1001.8570	2.93	4.03E-04	4.32E-04	5.14E-04	4.67E-04	4.54E-04	4.76E-05	1.33E-03	1.41E-03	2.12E-03	2.59E-03	1.87E-03	6.01E-04	2.93E-04	3.47E-04	4.21E-04	4.25E-04	3.71E-04	6.37E-05	1.55E-03	1.92E-03	1.81E-03	1.48E-03	1.69E-03	2.09E-04	3.28E-04	5.85E-04	6.62E-04	4.80E-04	5.14E-04	1.45E-04	0.003	0.08	0.00003	0.5	0.003	0.6	0.005	0.00002	0.1	0.00009
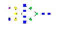	7-6-0-1	1122.2512	1122.2473	3.45	1.02E-02	9.84E-03	1.10E-02	9.53E-03	1.02E-02	6.54E-04	9.30E-04	9.27E-03	9.45E-03	9.31E-03	7.24E-03	4.21E-03	1.50E-02	1.36E-02	1.24E-02	1.35E-02	1.36E-02	1.05E-03	1.43E-02	1.11E-02	1.16E-02	1.30E-02	1.25E-02	1.42E-03	7.96E-04	8.90E-04	6.84E-04	7.99E-04	7.92E-04	8.44E-05	0.2	0.001	0.02	0	0.03	0.06	0.02	0.3	0	3E-06
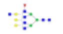	7-6-1-0	1059.8897	1059.8858	3.65	1.57E-04	1.70E-04	1.01E-04	2.33E-04	1.65E-04	5.39E-05	2.05E-03	2.06E-03	3.42E-03	4.38E-03	2.98E-03	1.13E-03	3.25E-04	4.98E-04	5.02E-04	5.53E-04	4.70E-04	9.97E-05	3.73E-03	2.64E-03	3.05E-03	2.58E-03	3.00E-03	5.31E-04	3.33E-04	4.22E-04	4.67E-04	4.32E-04	4.13E-04	5.72E-05	0.003	0.002	0.00004	0.0007	0.005	1	0.004	0.00008	0.4	0.00007
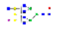	7-6-1-1	1180.2809	1180.2769	3.39	2.84E-03	2.38E-03	3.02E-03	3.70E-03	2.99E-03	5.49E-04	4.59E-03	5.63E-03	5.22E-03	5.90E-03	5.34E-03	5.71E-04	5.52E-03	6.76E-03	5.88E-03	6.42E-03	6.15E-03	5.49E-04	4.44E-03	8.10E-03	8.67E-03	8.01E-03	7.31E-03	1.93E-03	2.72E-03	3.49E-03	2.66E-03	2.90E-03	2.94E-03	3.76E-04	0.001	0.0002	0.005	0.9	0.09	0.1	0.0004	0.3	0.00007	0.004
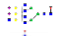	7-6-1-2	1300.6721	1300.6680	3.18	3.53E-03	3.26E-03	8.25E-04	3.47E-03	2.77E-03	1.30E-03	7.16E-04	8.03E-04	1.58E-04	3.22E-04	5.00E-04	3.09E-04	8.07E-04	2.29E-03	1.55E-03	2.39E-03	1.76E-03	7.36E-04	5.48E-05	8.32E-04	7.04E-04	6.62E-04	5.63E-04	3.47E-04	2.08E-04	3.02E-04	4.24E-04	4.83E-04	3.54E-04	1.23E-04	0.01	0.2	0.02	0.01	0.02	0.8	0.4	0.03	0.009	0.3
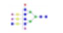	7-6-1-3	1421.0634	1421.0582	3.64	1.42E-03	1.29E-03	1.21E-04	6.52E-04	8.72E-04	6.04E-04	5.33E-04	5.27E-04	2.80E-04	3.36E-04	4.19E-04	1.30E-04	1.36E-04	7.10E-04	2.34E-04	7.58E-04	4.60E-04	3.20E-04	7.53E-05	1.34E-03	1.28E-03	2.24E-03	1.23E-03	8.86E-04	1.08E-04	1.66E-04	3.28E-04	1.08E-03	4.21E-04	4.50E-04	0.2	0.3	0.5	0.3	0.8	0.1	1	0.2	0.9	0.2
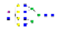	7-7-0-1	1190.2844	1190.2800	3.72	1.59E-03	1.45E-03	8.69E-04	2.04E-03	1.49E-03	4.84E-04	1.97E-03	2.17E-03	1.86E-03	1.67E-03	1.92E-03	2.09E-04	1.94E-03	2.28E-03	1.97E-03	2.26E-03	2.11E-03	1.83E-04	5.04E-04	1.24E-03	1.42E-03	1.34E-03	1.13E-03	4.22E-04	3.79E-04	5.34E-04	4.61E-04	6.81E-04	5.14E-04	1.28E-04	0.2	0.05	0.3	0.008	0.2	0.02	0.00003	0.005	7E-06	0.03
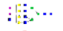	7-7-0-2	1310.6757	1310.6729	2.11	1.02E-03	8.77E-04	2.45E-04	8.41E-04	7.47E-04	3.44E-04	5.46E-04	5.34E-04	2.84E-04	4.98E-04	4.66E-04	1.23E-04	2.18E-04	4.77E-04	3.06E-04	5.57E-04	3.89E-04	1.55E-04	2.85E-05	1.68E-04	1.71E-04	1.93E-04	1.40E-04	7.53E-05	9.47E-05	2.34E-04	2.78E-04	4.88E-04	2.74E-04	1.63E-04	0.2	0.1	0.01	0.05	0.5	0.004	0.1	0.03	0.3	0.2
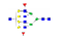	7-7-1-0	1127.9229	1127.9198	2.78	2.06E-03	1.75E-03	2.83E-03	2.72E-03	2.34E-03	5.23E-04	3.96E-03	4.59E-03	5.95E-03	7.09E-03	5.40E-03	1.40E-03	1.75E-03	1.66E-03	1.89E-03	1.72E-03	1.76E-03	9.48E-05	3.53E-03	3.29E-03	3.26E-03	2.72E-03	3.20E-03	3.45E-04	1.30E-03	1.64E-03	1.02E-03	1.25E-03	1.30E-03	2.56E-04	0.006	0.07	0.03	0.01	0.002	0.02	0.001	0.0002	0.02	0.0001
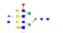	7-7-1-1	1248.3142	1248.3114	2.22	3.23E-03	2.70E-03	1.49E-03	3.41E-03	2.71E-03	8.64E-04	2.95E-03	3.72E-03	2.58E-03	2.88E-03	3.03E-03	4.87E-04	9.97E-04	1.54E-03	1.34E-03	1.73E-03	1.40E-03	3.13E-04	8.41E-04	3.24E-03	3.24E-03	2.91E-03	2.56E-03	1.15E-03	9.43E-04	1.41E-03	1.25E-03	1.58E-03	1.29E-03	2.72E-04	0.5	0.03	0.8	0.02	0.001	0.5	0.0008	0.1	0.6	0.08
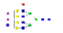	7-7-1-2	1368.7054	1368.7008	3.36	1.33E-02	6.05E-03	7.18E-04	4.84E-03	6.23E-03	5.24E-03	3.94E-03	5.00E-03	1.33E-03	2.02E-03	3.07E-03	1.69E-03	7.77E-04	2.77E-03	1.20E-03	2.77E-03	1.88E-03	1.04E-03	1.24E-04	2.25E-03	2.09E-03	2.93E-03	1.85E-03	1.21E-03	6.33E-04	1.20E-03	1.49E-03	3.09E-03	1.61E-03	1.05E-03	0.3	0.2	0.2	0.1	0.3	0.3	0.2	1	0.7	0.8
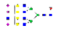	7-7-1-3	1489.0966	1489.0916	3.38	3.43E-04	5.02E-04	4.97E-05	3.10E-04	3.01E-04	1.87E-04	5.19E-04	5.44E-04	1.63E-04	1.74E-04	3.50E-04	2.10E-04	3.08E-05	1.16E-04	4.09E-05	9.92E-05	7.18E-05	4.23E-05	6.63E-06	1.37E-04	1.29E-04	3.01E-04	1.43E-04	1.21E-04	3.50E-05	4.24E-05	4.21E-05	2.13E-04	8.32E-05	8.69E-05	0.7	0.05	0.2	0.08	0.04	0.1	0.06	0.3	0.8	0.5
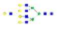	7-8-0-0	1137.9265	1137.9218	4.10	2.08E-03	1.59E-03	1.70E-03	1.93E-03	1.83E-03	2.20E-04	2.47E-03	2.98E-03	2.60E-03	2.55E-03	2.65E-03	2.24E-04	1.02E-03	1.11E-03	1.05E-03	1.18E-03	1.09E-03	7.12E-05	4.47E-04	5.57E-04	5.22E-04	4.76E-04	5.00E-04	4.89E-05	1.46E-04	1.87E-04	2.31E-04	2.15E-04	1.95E-04	3.72E-05	0.002	0.0007	0.00002	6E-06	0.00001	1E-06	1E-06	0.00001	1E-06	0.00006
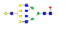	7-8-1-0	1195.9562	1195.9522	3.34	4.24E-03	3.81E-03	3.51E-03	4.36E-03	3.98E-03	3.95E-04	4.86E-03	5.59E-03	4.99E-03	5.98E-03	5.35E-03	5.23E-04	2.49E-03	2.31E-03	2.46E-03	2.47E-03	2.43E-03	8.07E-05	1.17E-03	2.49E-03	2.07E-03	1.92E-03	1.91E-03	5.52E-04	6.61E-04	7.17E-04	6.37E-04	7.24E-04	6.85E-04	4.24E-05	0.006	0.0003	0.0009	3E-06	0.00003	0.0001	2E-06	0.1	0	0.004
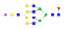	7-8-1-1	1316.3474	1316.3442	2.46	2.89E-03	2.38E-03	7.79E-04	2.78E-03	2.21E-03	9.76E-04	2.56E-03	3.26E-03	1.70E-03	2.00E-03	2.38E-03	6.90E-04	9.41E-04	1.52E-03	1.09E-03	1.40E-03	1.24E-03	2.67E-04	2.04E-04	1.67E-03	1.39E-03	1.39E-03	1.16E-03	6.54E-04	4.91E-04	7.66E-04	7.55E-04	1.29E-03	8.25E-04	3.35E-04	0.8	0.1	0.1	0.04	0.02	0.04	0.007	0.8	0.1	0.4
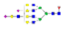	7-8-1-2	1436.7387	1436.7331	3.87	1.09E-03	9.95E-04	9.82E-05	9.09E-04	7.74E-04	4.57E-04	1.07E-03	1.19E-03	3.94E-04	4.53E-04	7.76E-04	4.11E-04	3.94E-05	1.81E-04	7.94E-05	1.91E-04	1.23E-04	7.52E-05	1.14E-05	3.53E-04	2.42E-04	5.85E-04	2.98E-04	2.39E-04	4.20E-05	8.92E-05	1.15E-04	5.70E-04	2.04E-04	2.46E-04	1	0.03	0.1	0.07	0.02	0.09	0.05	0.2	0.6	0.6
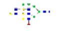	7-9-1-0	1263.9895	1263.9852	3.38	2.89E-03	2.18E-03	1.35E-03	3.16E-03	2.39E-03	8.09E-04	2.02E-03	2.76E-03	1.76E-03	2.32E-03	2.21E-03	4.28E-04	1.17E-03	1.19E-03	9.81E-04	1.23E-03	1.14E-03	1.11E-04	3.57E-04	1.09E-03	9.83E-04	9.44E-04	8.42E-04	3.29E-04	4.93E-04	5.85E-04	5.84E-04	7.42E-04	6.01E-04	1.03E-04	0.7	0.02	0.01	0.005	0.003	0.002	0.0003	0.1	0.0004	0.2
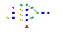	7-9-1-1	1384.3807	1384.3764	3.11	2.13E-03	1.68E-03	2.54E-04	2.25E-03	1.58E-03	9.16E-04	1.20E-03	1.67E-03	4.96E-04	6.26E-04	9.99E-04	5.44E-04	2.15E-04	5.55E-04	3.07E-04	6.15E-04	4.23E-04	1.92E-04	3.92E-05	7.90E-04	5.70E-04	8.74E-04	5.68E-04	3.75E-04	1.11E-04	2.10E-04	2.83E-04	6.63E-04	3.17E-04	2.41E-04	0.3	0.05	0.09	0.04	0.09	0.2	0.06	0.5	0.5	0.3
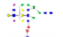	7-9-1-2	1504.7719	1504.7665	3.61	1.00E-03	1.16E-03	6.09E-04	1.11E-03	9.71E-04	2.50E-04	6.38E-04	7.92E-04	1.29E-04	1.68E-04	4.32E-04	3.34E-04	1.84E-05	6.88E-05	2.75E-05	7.19E-05	4.67E-05	2.76E-05	3.67E-06	9.02E-05	5.90E-05	2.08E-04	9.02E-05	8.62E-05	1.21E-05	2.37E-05	3.65E-05	2.57E-04	8.23E-05	1.17E-04	0.04	0.0003	0.0006	0.0007	0.06	0.09	0.1	0.4	0.6	0.9
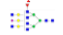	8-6-1-1	1261.9897	1261.9860	2.91	2.96E-04	2.67E-04	2.04E-04	3.49E-04	2.79E-04	6.04E-05	4.14E-04	4.80E-04	3.18E-04	5.00E-04	4.28E-04	8.21E-05	4.55E-04	4.96E-04	3.99E-04	4.88E-04	4.59E-04	4.41E-05	1.58E-04	3.37E-04	3.51E-04	2.09E-03	7.34E-04	9.08E-04	3.39E-05	3.12E-05	3.22E-05	4.37E-05	3.52E-05	5.73E-06	0.03	0.003	0.4	0.0002	0.5	0.5	0.00008	0.6	1E-06	0.2
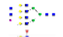	8-7-0-1	1271.9932	1271.9896	2.83	3.45E-04	3.33E-04	2.13E-04	4.00E-04	3.23E-04	7.89E-05	2.46E-04	3.09E-04	1.54E-04	1.89E-04	2.24E-04	6.78E-05	1.59E-04	1.80E-04	1.59E-04	2.18E-04	1.79E-04	2.76E-05	5.56E-05	1.55E-04	1.29E-04	1.41E-04	1.20E-04	4.43E-05	0.00E+00	0.00E+00	0.00E+00	0.00E+00	0.00E+00	0.00E+00	0.1	0.01	0.004	0.0002	0.3	0.04	0.0006	0.06	0.00001	0.002
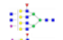	8-7-1-1	1330.0229	1330.0180	3.71	4.74E-04	4.28E-04	2.91E-04	5.17E-04	4.27E-04	9.81E-05	3.91E-04	5.37E-04	3.38E-04	5.50E-04	4.54E-04	1.06E-04	2.09E-04	3.22E-04	2.86E-04	2.81E-04	2.75E-04	4.75E-05	1.04E-04	2.88E-04	1.83E-04	2.10E-04	1.96E-04	7.56E-05	6.97E-05	7.56E-05	2.29E-04	2.10E-04	1.46E-04	8.50E-05	0.7	0.03	0.01	0.005	0.02	0.007	0.004	0.1	0.04	0.4
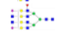	8-7-1-2	1450.4142	1450.4101	2.80	6.28E-04	4.58E-04	1.01E-05	7.00E-04	4.49E-04	3.10E-04	2.40E-04	2.46E-04	6.56E-05	1.51E-04	1.76E-04	8.53E-05	1.30E-04	4.08E-04	2.01E-04	3.88E-04	2.82E-04	1.38E-04	2.59E-05	4.47E-04	4.17E-04	4.76E-04	3.41E-04	2.12E-04	5.51E-05	9.22E-05	1.18E-04	2.53E-04	1.29E-04	8.61E-05	0.1	0.4	0.6	0.09	0.2	0.2	0.5	0.7	0.1	0.1
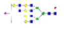	8-8-1-1	1398.0562	1398.0534	2.00	7.42E-04	6.94E-04	2.26E-04	9.24E-04	6.46E-04	2.98E-04	4.49E-04	6.34E-04	2.11E-04	3.59E-04	4.13E-04	1.77E-04	2.62E-04	4.82E-04	3.46E-04	4.37E-04	3.82E-04	9.77E-05	1.13E-04	3.03E-04	2.78E-04	2.73E-04	2.42E-04	8.67E-05	1.29E-04	2.10E-04	1.87E-04	2.19E-04	1.86E-04	4.05E-05	0.2	0.1	0.04	0.02	0.8	0.1	0.05	0.08	0.01	0.3
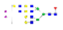	8-8-1-2	1518.4474	1518.4426	3.18	6.43E-04	6.77E-04	3.34E-05	7.19E-04	5.18E-04	3.25E-04	1.99E-04	2.91E-04	3.71E-05	7.20E-05	1.50E-04	1.17E-04	5.23E-05	2.22E-04	7.74E-05	1.89E-04	1.35E-04	8.28E-05	5.10E-06	2.30E-04	1.77E-04	3.56E-04	1.92E-04	1.46E-04	2.00E-05	3.17E-05	6.93E-05	2.40E-04	9.02E-05	1.02E-04	0.08	0.06	0.1	0.05	0.8	0.7	0.5	0.5	0.5	0.3
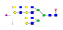	8-9-1-1	1466.0895	1466.0842	3.59	4.19E-04	3.53E-04	5.25E-05	4.70E-04	3.24E-04	1.87E-04	2.32E-04	3.49E-04	6.60E-04	1.23E-04	3.41E-04	2.32E-04	7.20E-05	1.49E-04	7.21E-05	1.18E-04	1.03E-04	3.76E-05	8.90E-06	1.21E-04	1.09E-04	1.18E-04	8.92E-05	5.38E-05	2.91E-05	3.73E-05	7.52E-05	1.43E-04	7.12E-05	5.21E-05	0.9	0.06	0.05	0.04	0.09	0.08	0.06	0.7	0.4	0.6
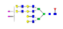	8-9-1-2	1586.4807	1586.4757	3.15	6.77E-04	6.03E-04	2.89E-05	7.39E-04	5.12E-04	3.27E-04	1.62E-04	2.19E-04	2.88E-05	5.10E-05	1.15E-04	9.03E-05	4.53E-05	1.64E-04	6.52E-05	1.72E-04	1.12E-04	6.56E-05	0.00E+00	0.00E+00	0.00E+00	0.00E+00	0.00E+00	0.00E+00	0.00E+00	0.00E+00	0.00E+00	0.00E+00	0.00E+00	0.00E+00	0.06	0.05	0.02	0.02	1	0.04	0.04	0.01	0.01	#DIV/0!

Table S2. List of Statistically Significant *N* -Glycans in proteinuria and hypertension male group (G3) and male control group (G4), Obese and diabetic male group (G5) and male control group (G4), proteinuria female group (G1) and female control group (G2), and female control group (G2) and male control group (G4) and their relative abundance with p-value.

Composition: X-X-X-X stands for HexNAc-Hexose-DeoxyHex-NeuAc. HexNAc includes N-acetylglucosamine and N-acetylgalactosamine. Hexose includes galactose and mannose. Deoxyhexose is fucose and NeuAc is N-acetylneuraminic acid.

Symbols: , -acetylglucosamine (GlcNAc); , Galacto (Gal); , Fucose (Fuc); , Mannose (Man); , N-acetylneuraminic aci (NeuAc/Sialic Acid),

Statistically Significant *N* -Glycans in male group with proteinuria and hypertension (G3) and male control group (G4)

Glycan	Composition	G3 33A	G3 33I	G3 34C	G3 35D	Ave relative Abu	Standard Deviati	G4 46A	G4 411	G4 481	G4 487	Ave relative Abu	Standard Deviatio	P-value(Bonferroni correction)
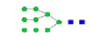	2-9-0-0	2.71E-02	2.20E-02	2.10E-02	2.20E-02	2.30E-02	2.77E-03	1.22E-02	1.31E-02	1.21E-02	1.15E-02	1.22E-02	6.53E-04	0.0003
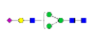	3-4-0-1	5.33E-03	4.66E-03	5.82E-03	4.37E-03	5.04E-03	6.54E-04	2.25E-03	2.30E-03	2.21E-03	2.25E-03	2.25E-03	3.77E-05	0.0001
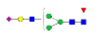	3-4-1-1	2.73E-03	2.45E-03	2.64E-03	2.02E-03	2.46E-03	3.16E-04	1.12E-03	1.23E-03	9.73E-04	1.05E-03	1.09E-03	1.10E-04	0.0002
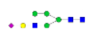	3-5-0-1	5.44E-03	5.27E-03	5.43E-03	4.84E-03	5.24E-03	2.83E-04	3.53E-03	3.71E-03	3.47E-03	3.69E-03	3.60E-03	1.21E-04	0.00004
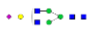	4-4-0-1	4.48E-03	3.87E-03	4.40E-03	3.99E-03	4.18E-03	2.99E-04	2.18E-03	2.31E-03	2.02E-03	2.39E-03	2.23E-03	1.61E-04	0.00003
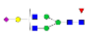	4-4-1-1	5.27E-03	5.48E-03	5.38E-03	4.89E-03	5.26E-03	2.57E-04	3.43E-03	3.52E-03	3.30E-03	4.11E-03	3.59E-03	3.60E-04	0.0003
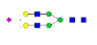	4-5-0-1	4.63E-03	4.63E-03	5.11E-03	4.59E-03	4.74E-03	2.45E-04	1.32E-03	2.19E-03	1.94E-03	2.29E-03	1.93E-03	4.35E-04	0.00003
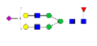	4-5-1-1	5.00E-03	5.87E-03	6.61E-03	5.12E-03	5.65E-03	7.43E-04	2.01E-03	2.45E-03	2.53E-03	2.64E-03	2.41E-03	2.77E-04	0.0002
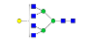	6-4-0-0	1.42E-03	1.38E-03	1.71E-03	1.44E-03	1.49E-03	1.49E-04	5.41E-03	3.76E-03	4.23E-03	3.76E-03	4.29E-03	7.77E-04	0.0004
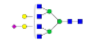	6-5-0-1	3.22E-03	3.67E-03	3.47E-03	3.75E-03	3.53E-03	2.37E-04	6.86E-03	5.99E-03	6.33E-03	5.75E-03	6.23E-03	4.84E-04	0.00006
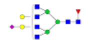	6-5-1-1	3.67E-03	4.19E-03	4.46E-03	4.37E-03	4.17E-03	3.54E-04	9.65E-03	1.11E-02	1.18E-02	9.15E-03	1.04E-02	1.23E-03	0.00007
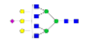	6-6-0-1	1.61E-03	1.82E-03	1.50E-03	1.56E-03	1.62E-03	1.40E-04	3.76E-03	5.37E-03	5.17E-03	4.95E-03	4.81E-03	7.23E-04	0.0001
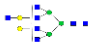	7-5-0-0	2.83E-04	3.28E-04	3.40E-04	3.33E-04	3.21E-04	2.58E-05	1.21E-03	1.46E-03	1.37E-03	1.64E-03	1.42E-03	1.76E-04	0.00002
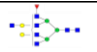	7-5-1-0	9.38E-04	1.31E-03	1.24E-03	1.41E-03	1.22E-03	2.02E-04	4.29E-03	3.97E-03	4.19E-03	3.14E-03	3.90E-03	5.23E-04	0.00008
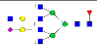	7-5-1-1	8.49E-04	9.26E-04	9.08E-04	1.11E-03	9.48E-04	1.14E-04	3.07E-03	3.29E-03	4.01E-03	2.92E-03	3.32E-03	4.83E-04	0.00007
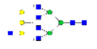	7-6-0-0	2.93E-04	3.47E-04	4.21E-04	4.25E-04	3.71E-04	6.37E-05	1.55E-03	1.92E-03	1.81E-03	1.48E-03	1.69E-03	2.09E-04	0.00002
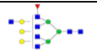	7-6-1-0	3.25E-04	4.98E-04	5.02E-04	5.53E-04	4.70E-04	9.97E-05	3.73E-03	2.64E-03	3.05E-03	2.58E-03	3.00E-03	5.31E-04	0.00008
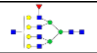	7-7-1-0	1.75E-03	1.66E-03	1.89E-03	1.72E-03	1.76E-03	9.48E-05	3.53E-03	3.29E-03	3.26E-03	2.72E-03	3.20E-03	3.45E-04	0.0002
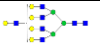	7-8-0-0	1.02E-03	1.11E-03	1.05E-03	1.18E-03	1.09E-03	7.12E-05	4.47E-04	5.57E-04	5.22E-04	4.76E-04	5.00E-04	4.89E-05	0.00001

**Statistically Significant *N*-Glycans in male group with obesity and diabetes (G5) and male control group (G4)**													
Glycan	Composition	G4 46A	G4 411	G4 481	G4 487	Ave relative Abu	Standard Deviati	G5 5F1	G5 5F2	G5 5F3	G5 5F4	Ave relative Abu	Standard Deviatio	P-value(Bonferroni correction)
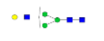	3-4-0-0	5.18E-04	4.11E-04	3.94E-04	3.73E-04	4.24E-04	6.44E-05	9.53E-04	1.00E-03	1.07E-03	1.14E-03	1.04E-03	8.21E-05	0.00002
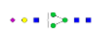	3-4-0-1	2.25E-03	2.30E-03	2.21E-03	2.25E-03	2.25E-03	3.77E-05	3.02E-03	3.46E-03	3.12E-03	3.11E-03	3.18E-03	1.94E-04	0.00008
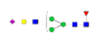	3-4-1-1	1.12E-03	1.23E-03	9.73E-04	1.05E-03	1.09E-03	1.10E-04	2.66E-03	2.78E-03	2.77E-03	3.37E-03	2.90E-03	3.18E-04	0.00004
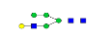	3-5-0-0	3.61E-03	3.24E-03	2.93E-03	3.02E-03	3.20E-03	3.03E-04	6.26E-03	6.03E-03	6.73E-03	7.66E-03	6.67E-03	7.22E-04	0.0001
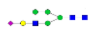	3-5-0-1	3.53E-03	3.71E-03	3.47E-03	3.69E-03	3.60E-03	1.21E-04	4.75E-03	4.63E-03	5.20E-03	4.73E-03	4.83E-03	2.52E-04	0.0001
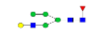	3-5-1-0	1.06E-03	9.61E-04	8.05E-04	8.18E-04	9.11E-04	1.22E-04	3.89E-03	3.36E-03	4.18E-03	5.04E-03	4.12E-03	6.99E-04	0.0001
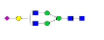	4-4-0-1	2.18E-03	2.31E-03	2.02E-03	2.39E-03	2.23E-03	1.61E-04	3.26E-03	3.19E-03	3.52E-03	3.42E-03	3.35E-03	1.50E-04	0.00005
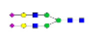	4-5-0-2	4.34E-03	7.17E-03	9.60E-03	1.11E-02	8.06E-03	2.97E-03	2.06E-02	2.41E-02	2.74E-02	2.51E-02	2.43E-02	2.84E-03	0.0002
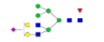	4-7-1-1	3.29E-05	1.21E-04	1.34E-04	9.58E-05	9.60E-05	4.50E-05	3.11E-04	3.49E-04	4.24E-04	4.19E-04	3.76E-04	5.49E-05	0.0002
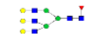	5-6-1-0	5.57E-03	6.59E-03	5.80E-03	5.94E-03	5.98E-03	4.37E-04	7.89E-03	7.54E-03	8.17E-03	7.98E-03	7.89E-03	2.66E-04	0.0003
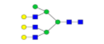	5-7-0-0	1.69E-03	2.14E-03	2.09E-03	1.89E-03	1.95E-03	2.05E-04	4.16E-03	3.44E-03	4.18E-03	4.39E-03	4.04E-03	4.16E-04	0.0001
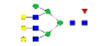	5-7-1-0	7.21E-03	1.01E-02	9.96E-03	8.27E-03	8.89E-03	1.40E-03	1.87E-02	1.69E-02	1.91E-02	2.01E-02	1.87E-02	1.36E-03	0.00006
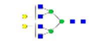	6-5-0-0	1.02E-02	7.44E-03	7.14E-03	7.47E-03	8.06E-03	1.43E-03	2.44E-03	2.83E-03	1.92E-03	2.47E-03	2.42E-03	3.75E-04	0.0003
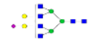	6-5-0-1	6.86E-03	5.99E-03	6.33E-03	5.75E-03	6.23E-03	4.84E-04	2.29E-03	2.24E-03	2.11E-03	1.94E-03	2.14E-03	1.57E-04	0.000004
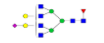	6-5-1-1	9.65E-03	1.11E-02	1.18E-02	9.15E-03	1.04E-02	1.23E-03	6.15E-03	5.91E-03	5.66E-03	4.93E-03	5.66E-03	5.29E-04	0.0004
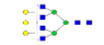	6-6-0-0	4.52E-03	4.18E-03	3.88E-03	3.84E-03	4.11E-03	3.17E-04	2.46E-03	2.82E-03	2.59E-03	2.63E-03	2.63E-03	1.48E-04	0.0001
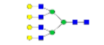	6-7-0-0	5.53E-03	4.76E-03	4.86E-03	4.61E-03	4.94E-03	4.09E-04	2.31E-03	2.33E-03	2.00E-03	2.30E-03	2.24E-03	1.55E-04	0.00002
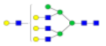	6-8-0-0	6.05E-04	6.82E-04	6.91E-04	6.48E-04	6.57E-04	3.89E-05	3.75E-04	3.93E-04	3.53E-04	4.24E-04	3.86E-04	3.01E-05	0.00003
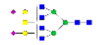	7-5-0-2	7.35E-04	6.56E-04	7.55E-04	5.59E-04	6.76E-04	8.91E-05	2.67E-04	3.34E-04	3.42E-04	3.42E-04	3.22E-04	3.66E-05	0.0003
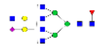	7-5-1-1	3.07E-03	3.29E-03	4.01E-03	2.92E-03	3.32E-03	4.83E-04	1.22E-04	2.18E-04	1.21E-04	1.41E-04	1.51E-04	4.61E-05	0.00001
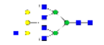	7-6-0-0	1.55E-03	1.92E-03	1.81E-03	1.48E-03	1.69E-03	2.09E-04	3.28E-04	5.85E-04	6.62E-04	4.80E-04	5.14E-04	1.45E-04	0.00009
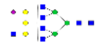	7-6-0-1	1.43E-02	1.11E-02	1.16E-02	1.30E-02	1.25E-02	1.42E-03	7.96E-04	8.90E-04	8.90E-04	8.90E-04	7.92E-04	8.44E-05	0.000003
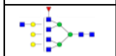	7-6-1-0	3.73E-03	2.64E-03	3.05E-03	2.58E-03	3.00E-03	5.31E-04	3.33E-04	4.22E-04	4.67E-04	4.32E-04	4.13E-04	5.72E-05	0.00007
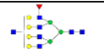	7-7-1-0	3.53E-03	3.29E-03	3.26E-03	2.72E-03	3.20E-03	3.45E-04	1.30E-03	1.64E-03	1.02E-03	1.25E-03	1.30E-03	2.56E-04	0.0001
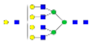	7-8-0-0	4.47E-04	5.57E-04	5.22E-04	4.76E-04	5.00E-04	4.89E-05	1.46E-04	1.87E-04	2.31E-04	2.15E-04	1.95E-04	3.72E-05	0.00006

Statistically Significant *N*-Glycans in female group with proteinuria (G1) and female control group (G2)													
Glycan	Composition	G1 12A	G1 13G	G1 12Z	G1 113	Ave relative Abu	Standard Deviati	G2 23A	G2 23B	G2 26B	G2 26D	Ave relative Abur	Standard Deviati	P-value(Bonferroni correction)
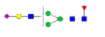	3-4-1-1	1.32E-03	1.36E-03	1.49E-03	9.46E-04	1.28E-03	2.33E-04	4.17E-03	4.40E-03	4.80E-03	3.53E-03	4.23E-03	5.29E-04	0.00005
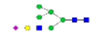	3-6-0-1	7.25E-03	7.24E-03	6.75E-03	6.08E-03	6.83E-03	5.56E-04	4.63E-03	4.72E-03	4.55E-03	4.59E-03	4.62E-03	7.35E-05	0.0002
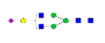	4-4-0-1	2.83E-03	2.74E-03	2.96E-03	1.78E-03	2.58E-03	5.41E-04	7.83E-03	7.53E-03	7.83E-03	7.59E-03	7.70E-03	1.58E-04	0.000002
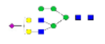	4-6-0-1	1.10E-02	9.10E-03	7.87E-03	8.65E-03	9.16E-03	1.34E-03	4.08E-03	4.48E-03	3.72E-03	3.61E-03	3.97E-03	3.95E-04	0.0003
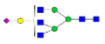	5-4-0-1	4.57E-02	4.41E-02	4.42E-02	4.26E-02	4.41E-02	1.24E-03	8.50E-02	7.89E-02	7.37E-02	7.34E-02	7.78E-02	5.41E-03	0.00002
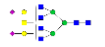	7-5-0-2	8.10E-04	7.99E-04	6.29E-04	6.94E-04	7.33E-04	8.67E-05	1.66E-04	1.62E-04	8.23E-05	1.32E-04	1.36E-04	3.86E-05	0.00002

Statistically Significant *N*-Glycans in female control group (G2) and male control group (G4)													
Glycan	Composition	G2 23A	G2 23B	G2 26B	G2 26D	Ave relative Abu	Standard Deviati	G4 46A	G4 411	G4 481	G4 487	Ave relative Abur	Standard Deviati	P-value(Bonferroni correction)
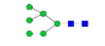	2-6-0-0	8.80E-02	8.94E-02	9.63E-02	7.58E-02	8.74E-02	8.52E-03	5.64E-02	5.39E-02	5.27E-02	5.30E-02	5.40E-02	1.70E-03	0.0003
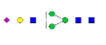	3-4-0-1	1.31E-03	1.29E-03	1.14E-03	1.36E-03	1.27E-03	9.54E-05	2.25E-03	2.30E-03	2.21E-03	2.25E-03	2.25E-03	3.77E-05	0.000001
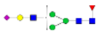	3-4-1-1	4.17E-03	4.40E-03	4.80E-03	3.53E-03	4.23E-03	5.29E-04	1.12E-03	1.23E-03	9.73E-04	1.05E-03	1.09E-03	1.10E-04	0.00002
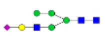	3-5-0-1	2.33E-03	2.13E-03	2.32E-03	2.37E-03	2.29E-03	1.06E-04	3.53E-03	3.71E-03	3.47E-03	3.69E-03	3.60E-03	1.21E-04	0.000003
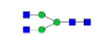	4-3-0-0	4.10E-03	2.67E-03	2.79E-03	4.19E-03	3.44E-03	8.17E-04	1.10E-02	9.36E-03	8.41E-03	8.70E-03	9.37E-03	1.18E-03	0.0002
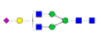	4-4-0-1	7.83E-03	7.53E-03	7.83E-03	7.59E-03	7.70E-03	1.58E-04	2.18E-03	2.31E-03	2.02E-03	2.39E-03	2.23E-03	1.61E-04	5.E-09
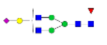	4-4-1-1	1.64E-03	1.82E-03	1.75E-03	1.59E-03	1.70E-03	1.06E-04	3.43E-03	3.52E-03	3.30E-03	4.11E-03	3.59E-03	3.60E-04	0.00006
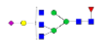	5-4-1-1	3.88E-02	3.75E-02	3.98E-02	4.21E-02	3.96E-02	1.94E-03	8.94E-02	7.39E-02	7.78E-02	7.62E-02	7.93E-02	6.94E-03	0.00003
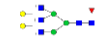	5-5-1-0	6.39E-02	5.68E-02	5.82E-02	5.98E-02	5.97E-02	3.10E-03	4.19E-02	3.93E-02	3.70E-02	3.77E-02	3.90E-02	2.18E-03	0.00003
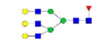	5-6-1-0	1.35E-02	1.29E-02	1.38E-02	1.16E-02	1.29E-02	9.89E-04	5.57E-03	6.59E-03	5.80E-03	5.94E-03	5.98E-03	4.37E-04	0.00001
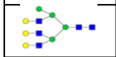	5-7-0-0	3.45E-03	3.39E-03	3.31E-03	2.79E-03	3.24E-03	3.01E-04	1.69E-03	2.14E-03	2.09E-03	1.89E-03	1.95E-03	2.05E-04	0.0004
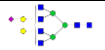	6-5-0-1	2.94E-03	2.87E-03	3.41E-03	2.95E-03	3.04E-03	2.49E-04	6.86E-03	5.99E-03	6.33E-03	5.75E-03	6.23E-03	4.84E-04	0.00002
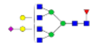	6-5-1-1	2.67E-03	2.41E-03	2.60E-03	3.37E-03	2.76E-03	4.21E-04	9.65E-03	1.11E-02	1.18E-02	9.15E-03	1.04E-02	1.23E-03	0.00002
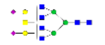	7-5-0-2	1.66E-04	1.62E-04	8.23E-05	1.32E-04	1.36E-04	3.86E-05	7.35E-04	6.56E-04	7.55E-04	5.59E-04	6.76E-04	8.91E-05	0.00003
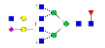	7-5-1-1	5.09E-04	5.33E-04	4.84E-04	7.74E-04	5.75E-04	1.34E-04	3.07E-03	3.29E-03	4.01E-03	2.92E-03	3.32E-03	4.83E-04	0.00003
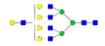	7-8-0-0	2.47E-03	2.98E-03	2.60E-03	2.55E-03	2.65E-03	2.24E-04	4.47E-04	5.57E-04	5.22E-04	4.76E-04	5.00E-04	4.89E-05	0.000001
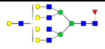	7-8-1-0	4.86E-03	5.59E-03	4.99E-03	5.98E-03	5.35E-03	5.23E-04	1.17E-03	2.49E-03	2.07E-03	1.92E-03	1.91E-03	5.52E-04	0.0001
